# On the evaluation of android malware detectors against code-obfuscation techniques

**DOI:** 10.7717/peerj-cs.1002

**Published:** 2022-06-21

**Authors:** Umair Nawaz, Muhammad Aleem, Jerry Chun-Wei Lin

**Affiliations:** 1 Computer Sciences, National University of Computer and Emerging Sciences, Islamabad, Islamabad, Pakistan; 2 Computer Sciences, Western Norway University of Applied Sciences, Bergen, Norway

**Keywords:** Android, Android’s anti-malware system, Obfuscation techniques, Reverse engineering

## Abstract

The Android mobile platform is the most popular and dominates the cell phone market. With the increasing use of Android, malware developers have become active in circumventing security measures by using various obfuscation techniques. The obfuscation techniques are used to hide the malicious code in the Android applications to evade detection by anti-malware tools. Some attackers use the obfuscation techniques in isolation, while some attackers use a mixed approach (*i.e*., employing multiple obfuscation techniques simultaneously). Therefore, it is crucial to analyze the impact of the different obfuscation techniques, both when they are used in isolation and when they are combined as hybrid techniques. Several studies have suggested that the obfuscation techniques may be more effective when used in a mixed pattern. However, in most of the related works, the obfuscation techniques used for analysis are either based on individual or a combination of primitive obfuscation techniques. In this work, we provide a comprehensive evaluation of anti-malware tools to gauge the impact of complex hybrid code-obfuscations techniques on malware detection capabilities of the prominent anti-malware tools. The evaluation results show that the inter-category-wise hybridized code obfuscation results in more evasion as compared to the individual or simple hybridized code obfuscations (using multiple and similar code obfuscations) which most of the existing related work employed for the evaluation. Obfuscation techniques significantly impact the detection rate of any anti-malware tool. The remarkable result *i.e*., almost 100% best detection rate is observed for the seven out of 10 tools when analyzed using the individual obfuscation techniques, four out of 10 tools on category-wise obfuscation, and not a single anti-malware tool attained full detection (*i.e*., 100%) for inter-category obfuscations.

## Introduction

**Figure 1:**
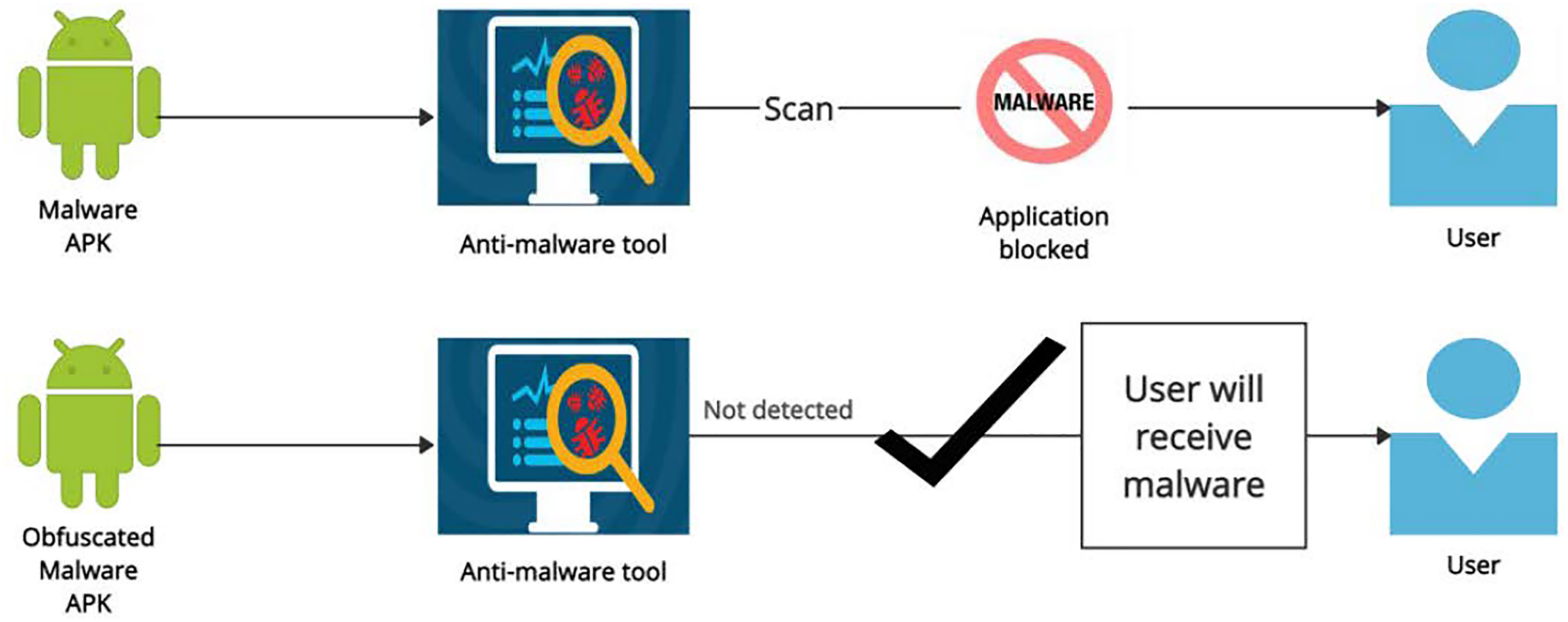
Malware *vs* obfuscated malware.

Google Play was originally launched in October 2008 (Threatpost, 2020). Android smartphones are among the most sought-after handheld devices around the world. Millions of apps are supported on these devices. According to Statista (2021), Android has become the most employed platform in the world with approximately 1999.12 million users worldwide. The increasing number of applications in the PlayStore makes Android smartphone users an attractive target too for cyber-criminals. Google has stepped up its efforts to ban malicious apps potentially being hosted on Google Play. In several other studies, researchers found that malicious or unwanted apps have their presence even on the Google Play Store. The number of new apps for Android devices decreased slightly in the first half of 2018. Even Google has taken measures to protect Android users from malware; however, there are numerous mechanisms employed by malware developers to bypass security measures. Malware authors are always looking for new tricks and mechanisms to bypass Android security. The malware authors adopt different obfuscation and transformation techniques to bypass the anti-malware detection (Elsersy, Feizollah & Anuar, 2022). The main motivation of this work is to evaluate the anti-malware tools against the malware that uses code-obfuscations. As shown in Fig. 1, the malware APK is blocked by the anti-malware tool after scan but when malware is obfuscated it is not detected by the anti-malware tool and the user will receive a malware. A large number of malware use obfuscation techniques to increase the evasion rate. There is a need to evaluate the anti-malware tools for complex obfuscation techniques.

With the largest market share of 85% (Statista, 2021), Android is dominating the mobile platform market and had become the most popular platform for mobile devices. The increase in popularity of the Android is because it is based on one of the most trusted and secure operating systems *i.e*., the Linux kernel, and that provides great performance and security. The Linux OS kernel is compatible with different hardware and can cut hardware costs too. Android is an open-source platform which means anyone can expand and inspect its source code considering the specific requirements. According to the researchers and application developers, Android is not only the most popular but also the most targeted platform too because of the huge number of Android-based devices worldwide.

Some of the significant threats on the Android platform (Bakour, Ünver & Ghanem, 2019) include ransomware, adware, trojan-spy, and SMS trojans. Most of the malware developers evade detection by applying different code obfuscations (Preda & Maggi, 2017; Hammad, Garcia & Malek, 2018) to hide the malicious code within the original APKs. Europe’s largest cyber-security services provider Kaspersky Lab estimated that in 2020, approximately 6 million attacks per month affected Android mobile devices (Chebyshev, 2021). Most of malicious applications hide the malicious code using different code obfuscations. A summary of similar studies is presented in Table 1. Most of the anti-malware tools use signature-based mechanisms to detect malware (Preda & Maggi, 2017). A signature can easily be evaded using various obfuscation techniques. Malware developers can increase the evasion rate by using a variety of obfuscation techniques (Preda & Maggi, 2017; Hammad, Garcia & Malek, 2018). Code obfuscation refers to code transformation to hide the code and execution patterns of the malware and produce an illusion of legitimate applications. In code obfuscation, the code is changed in such a manner that the program semantics remain the same. Malware authors use a wide range of obfuscation techniques to evade potential malicious activities. This study will provide researchers and developers of anti-malware tools with in-depth insight into the potential impact of different code-obfuscations and help them to assess the resilience of the prominent anti-malware tools against the potential threats based on code obfuscations.

**Table 1:** Malicious applications with total no. of downloads (Threatpost, 2020).

Harmful app type	Number of apps	Number of installs
Adware	48	300,600,000+
Subscription scam	15	20,000,000+
Hidden ads	57	14,550,000+
SMS premium subscription	24	472,000+

The work aims to provide a comprehensive study to evaluate anti-malware tools. This study employs various code-obfuscation techniques applied to the Android-based malware APKs to perform a comprehensive analysis of the prominent anti-malware tools. The obfuscation techniques are used in different ways, such as in isolation or individually, or in combination (from the same or different types of obfuscations) often referred to as hybrid obfuscation. Obfuscation techniques are applied in isolation, inter-category-wise, and inter-category to evaluate anti-malware tools. The main contributions of this work are as follows:

- Twenty different obfuscation techniques are used to evaluate prominent anti-malware tools;
- Obfuscation techniques are applied in isolation, category-wise, and inter-category-wise to evaluate anti-malware tools;
- Possible complex combinations that can be generated by applying multiple obfuscation categories simultaneously are used to evaluate anti-malware tools;
- The study has highlighted some of the significant hybridized code-obfuscations mechanisms which result in the highest evasion. The study provides a ground for future research for anti-malware tools developers and companies.

The remainder of the paper is as follows: “Related Work” discusses a literature review or related work. “Obfuscation Techniques and Working of Anti-malware Tools” provides background, discussing how anti-malware tools work and various obfuscation techniques. Then, in “Evaluating Anti-malware Tools”, the methodology for evaluating anti-malware tools is presented. The evaluation of the experiments and discussions are presented in “Experimental Evaluation”. The last “Conclusions and Future Work” is devoted to conclusions and future work.

## Related Work

Bakour, Ünver & Ghanem (2019) analyze the performance of various anti-malware tools, including Kaspersky (Kaspersky, 2022), McAfee (McAfee, 2022), Symantec (Symantec, 2021), ESET-NOD32 (ESET, 2022), Avira (Avira, 2022), and Dr. Web (Web, 2022), using different obfuscation techniques. These techniques are hybridized with the three different attacks proposed by the authors. These include the app *re-signing* attack, which replaces the original application signed with a new one by using a new certificate. The second attack proposed by the authors is the permission injection attack. In this, a list of all benign permissions is extracted from the manifest file and another malicious permission is injected into an application to evade detection by anti-malware tools. The third attack is permission code injection, where malicious code is injected into the application along with the permissions. Three different obfuscation techniques are used to create three different datasets (*i.e*., Refection Dataset, String Encryption Dataset, and Class Encryption Dataset). Then, these datasets are combined with the different attacks called app-resigning, permission injection attack, and permission-code injection attack. Different obfuscation techniques are mixed with different attacks. If the attacks are combined with only three different obfuscation techniques, a more diverse hybridization could have been used for the analysis.

Malware developers today are often able to evade malicious application detection through obfuscation (Preda & Maggi, 2017). Preda & Maggi (2017) have proposed an approach called Automatic Android Malware Obfuscator (AAMO). In this work, 17 different obfuscation techniques were used, including Android-specific, simple control flow modifications, advanced control flow modifications, renaming, and encryption, to name a few of the most important obfuscation aspects. Each original APK is passed to the APKTool for decompression, after which the obfuscation technique is applied using the obfuscation tool. After that, the APK is recompressed with the APKtool and re-signed with the tool jarsigner. This obfuscated APK is given to the anti-malware software for evaluation. For the evaluation, the authors used six anti-malware tools (*i.e*., Avast, Norton, Dr. Web, Kaspersky, Trend Micro, and Zoner). The obfuscation techniques are divided into categories and one of the category-based obfuscations was applied in each case. The authors did not provide an assessment with a hybrid assessment with multiple categories.

Hammad, Garcia & Malek (2018) use the technique of code obfuscation to evade detection by any anti-malware tool. They have developed a framework consisting of four modules: IR converter, IR transformer, APK generator, and data analyzer. The IR converter takes an APK file and converts the code into an Intermediate representation format. IR transformation applies obfuscation. IR generator repacks the obfuscated APK file, while the data Analyzer scans the APK and displays the results on whether the obfuscated APK is detected or not. The authors explained that anti-malware tools are slow to update their databases. If the databases are not up to date, signature-based detection becomes ineffective. The authors used different obfuscation techniques to evaluate the anti-malware tools, using only one of the obfuscation methods at a time.

Chua & Balachandran (2018) proposed an automated framework consisting of four obfuscation techniques (*i.e*., try-catch, method overloading, opaque predicate, and switch statement obfuscation) was developed. VirusTotal API is used to identify the malware samples. After applying obfuscation, a measure called *Escape Detection Rate* (EDR) was introduced. The EDR of more than 0.8 proves that signature-based detection is bypassed. The 57 anti-malware tools listed on VirusTotal were used for the evaluation. In this work, only occasional obfuscation techniques are used and only four basic techniques are used for the evaluation.

Aonzo et al. (2020) proposed an open-source tool Obfuscapk is used to apply obfuscation techniques. The Obfuscapk is an automated open-source tool for Android applications. An original APK file of any android is given for obfuscation, applying various obfuscation techniques and recreating the new APK file with a new signature and alignment. The newly generated APK file is encrypted once. In this work, five different obfuscation techniques are used. After applying the obfuscation, the obfuscated APK file is uploaded to VirusTotal for evaluation. In this work, the obfuscation techniques were mainly used in isolation and the effects of hybridizing the different obfuscation techniques on virus evasion were not studied.

Badhani & Muttoo (2019) divided obfuscation techniques into four levels to evaluate anti-malware tools. Level-A obfuscation techniques do not modify the source code of applications. Level-B includes all control flow-related obfuscation techniques. Level-C includes other obfuscation techniques such as renaming, *etc*., and Level-D includes encryption-related obfuscation techniques. The authors used three different automated obfuscator tools to obfuscate the app. Both single-level and multi-stage obfuscations are used to test the resistance of anti-malware tools.

The code reordering obfuscation technique is used by Cimitile et al. (2017) to make ransomware samples unrecognizable. The technique consists of two main steps. In the first step, a Java bytecode is compiled and code obfuscation (random reordering) is applied. The original execution order is preserved using goto statements. The second step is mainly concerned with verifying the obfuscation technique before using the example for evaluation. The Virustotal platform is used for the evaluation. This work uses only the simple obfuscation by reordering and ignores other potent obfuscation mechanisms.

Bacci et al. (2018) discuss the impact of obfuscation techniques on static and dynamic malware analysis. Eight different obfuscation techniques are used for the evaluation of anti-malware tools. For dynamic analysis, each application is executed on an Android mobile device for at least one minute to detect whether the application is malicious or not. The experiments confirm that the dynamic analysis-based detection is more robust to the code obfuscation techniques than the static analysis-based detection.

Li et al. (2019) proposed an approach called Obfusifier to identify the Android malware applications that have been transformed by various obfuscation techniques. An Android-platform-specific Java-based tool called *ALAN* is employed to apply code obfuscations. The tool ALAN supports seven different obfuscation techniques. After applying the obfuscation techniques, the virustotal platform is used to analyze the obfuscated malware samples. Various anti-malware tools from the virustotal platform are used. Along with the detection of obfuscated malware the other performance aspects such as the time taken for the analysis were also noted.

In the above literature as shown in Table 2, most of the related works (Preda & Maggi, 2017; Hammad, Garcia & Malek, 2018; Bakour, Ünver & Ghanem, 2019; Chua & Balachandran, 2018; Balachandran et al., 2016) use a limited number of obfuscation techniques and their combinations. In Chua & Balachandran (2018), only basic four obfuscation techniques (*i.e*., try-catch, method overloading, opaque predicate, and switch statement obfuscation) are used. However, the combinations of these techniques are not experimented with to evaluate the anti-malware tools. Although in Preda & Maggi (2017), 17 different obfuscation techniques are mentioned, these techniques are categorized and used to evaluate the anti-malware tools. However, the complex combinations (*i.e*., employing several inter-category techniques simultaneously) were not used for evaluation. In Hammad, Garcia & Malek (2018), 11 different obfuscation techniques are used sequentially (*i.e*., applying one at a time). In Bakour, Ünver & Ghanem (2019), three different obfuscation techniques are combined with the different attacking mechanism (*i.e*., app redesigning, permission injection attacks, and permission code injection attack, *etc*.) to increase the evasion rate. In some other related works (Hammad, Garcia & Malek, 2018; Chua & Balachandran, 2018), obfuscation techniques are used separately. There are few studies (Bakour, Ünver & Ghanem, 2019; Preda & Maggi, 2017) that use obfuscation techniques in a hybrid form to evaluate anti-malware tools. However, these techniques employ naive obfuscation techniques for the evaluation.

**Table 2:** Anti-malware evaluation techniques.

Ref.	Methodology/Approach	Limitations	Strengths
Chua & Balachandran (2018)	• An automated framework.	• Used four obfuscation techniques. -Each technique is used separately without any combination.	• An automated framework does obfuscation.
	• Four obfuscation techniques (*i.e*., try catch, method overloading, Opaque Predicate, and Switch Statement Obfuscation).		• Anti-malware tools that are listed on VirusTotal were used for evaluation.
	• VirusTotal API is used to classify the malware samples.		
	• EDR greater than 0.8 proved that signature-based detection is evaded.		
Preda & Maggi (2017)	• AAMO (Automatic Android Malware Obfuscator) framework.	• The same Obfuscation techniques are categorized.	• An automated framework AAMO does obfuscation.
	• 17 different Obfuscation techniques are used.	• A combination of any two categories is not used.	• 17 obfuscation techniques are listed on AAMO
	• Any original APK is given to APKTool for un-compression		
	• Then obfuscation technique is applied		
	• APK is then resigned with jarsigner.		
Bakour, Ünver & Ghanem (2019)	• Three different obfuscation techniques are used	• Attacks are combined with only three different obfuscation techniques.	• Various attacks are introduced with obfuscation techniques.
	• Three different datasets (*i.e*., Refection Dataset, String Encryption Dataset, Class Encryption Dataset)		
	• Various attacks named app-resigning, permission injection attack, and permission-code injection attack are used.		
Hammad, Garcia & Malek (2018)	• Framework based on four modules.	• Only one obfuscation technique is applied at a time.	• An automated framework with four different modules is used for applying obfuscation to applications.
	• IR converter takes an APK file and converts code into Intermediate representation format.		• This work shows that anti-malware tools are slow to update their databases.
	• IR transformation applies Obfuscation.		
	• IR generator repacks the obfuscated APK file.		
	• Data Analyzer scans the APK and shows results if the obfuscated APK is detected or not.		
Aonzo et al. (2020)	• Obfuscapk an obfuscation tool for android applications.	• All the work is automated.	• An automated framework is used for obfuscating the applications.
	• An original APK file is given to obfuscapk.	• Obfuscation techniques are applied through obfuscapk.	• VirusTotal API is used to evaluate the anti-malware tools.
	• Rebuild the new APK file with a new signature and new alignment.	• VirusTotal shows detection results.	
Balachandran et al. (2016)	• Seven different control flow techniques are used.	• Eight various automated obfuscation tools are used.	• Control flow obfuscation techniques are applied with various tools.
	• Dalvik bytecode to apply obfuscation techniques.	• Used two techniques at a time.	
	• The proposed approach preserves the execution order of the original instructions.		
	• New instructions to redirect to original instructions.		
Badhani & Muttoo (2019)	• Level-wise categorization of obfuscation techniques to test the resilience of anti-malware tools.	• All work is automated.	• Apply level wise obfuscation to test the resilience of different tools.
	• Level A: Obfuscations that do not alter the source code.	• Three automated obfuscators were used in experiments.	• Three different tools are used to apply obfuscation on different level.
	• Level B: Alter the code by changing the control flow.	• Three automated obfuscators were used in experiments.	
	• Level C: Perform renaming.	• level A is the easiest, and level D is the toughest to defeat.	
	• Level D: Perform encryption.		
Cimitile et al. (2017)	• Two primary processes are used	• Only ransomware samples are obfuscated.	• VirusTotal API is used to evaluate the anti-malware tools.
	• 1. Translation of Java bytecode.	• Code reordering obfuscation is used.	• Ransomware malware samples are obfuscation with simple obfuscation techniques.
	• 2. Investigate if the application is obfuscated or not.		• After obfuscation, the test is conducted to check if obfuscation techniques are applied successfully or not.
	• VirusTotal is used for the detection of malware samples.		
Bacci et al. (2018)	• Static and dynamic two different malware detection methods are used.	• Only eight Obfuscation techniques are used in an isolated way for analysis.	• These malware samples are installed on mobile devices to test the anti-malware tool’s resilience.
	• Eight different code obfuscations are used.		
	• Each application is executed on an android mobile device for at least one minute to detect if the application is malicious or not.		
Li et al. (2019)	• Obfusifier is introduced to identify the android malware applications.	• Obfuscate malicious applications by the ALAN tool.	• An automated framework used for obfuscation.
	• ALAN: A Java-based code obfuscation tool for Android is used for applying obfuscation techniques.	• Obfusifier cannot detect malicious applications which are obfuscated other than the ALAN tool.	• Anti-malware tools that are listed on VirusTotal were used for evaluation.
	• VirusTotal is used for the detection of malware samples.	• ALAN supports only seven different obfuscation techniques.	
Tang et al. (2022)	• AVPASS tool is used for obfuscation of any application.	• Obfuscation techniques are applied through AVPASS.	• Obfuscation techniques are applied through an automated framework.
	• AVPASS supports seven different obfuscation techniques.	• AVPASS only supports seven different obfuscation techniques.	• VirusTotal API is used for evaluation purposes.
	• Any original APK is given to AVPASS for obfuscation.		
	• After obfuscation, the application is assigned to Virustotal for detection of results.		

In summary, this work employs extensive obfuscation techniques (*i.e*., 20 potent techniques) along with the complex combinations of these obfuscations. The complex combinations are employed both category-wise (combining similar) or inter-category-wise (such as in the hybridized form) which most of the existing work lacks. Before producing the complex hybrid combinations, we initially grouped related obfuscation techniques. Using these obfuscation groups, we produced hybrid obfuscated malware samples by applying multiple obfuscation methods from a specific group. After that, the more complex hybrid obfuscations are applied to build malware samples. These complex hybrid mechanisms are based on multiple obfuscation techniques from different code-obfuscation groups (combining inter-category techniques) which most of the related work lacks.

## Obfuscation Techniques and Working of Anti-Malware Tools

Anti-malware tools use various mechanisms and techniques to analyze whether the file is malicious or benign (Al-Asli & Ghaleb, 2019). Anti-malware tools usually use three techniques for detection, namely signature-based, pattern-based, and heuristic-based models (Fang et al., 2017). The signature-based mechanisms (*i.e*., using malware signatures) are the most common method for detecting malicious files (Canfora et al., 2015). The signature-based method is the most used mechanism to detect malware. However, pattern-based malware detection is one of the most effective mechanisms. In pattern-based detection, the anti-malware tools use a specific code sequence to detect malicious applications (Malhotra & Bajaj, 2016). In heuristic-based analysis, the behavior of any application is monitored and related run-time information is employed for analysis (Alharbi et al., 2020). If the behavior of the file is suspicious or the application performs a certain suspicious activity, it is classified as malicious. Malware authors use many obfuscation techniques to perform malicious activities. Below are some of the most common obfuscation techniques employed by malware developers and Table 3 shows the impact factors of these obfuscation techniques.

**Figure 2:**
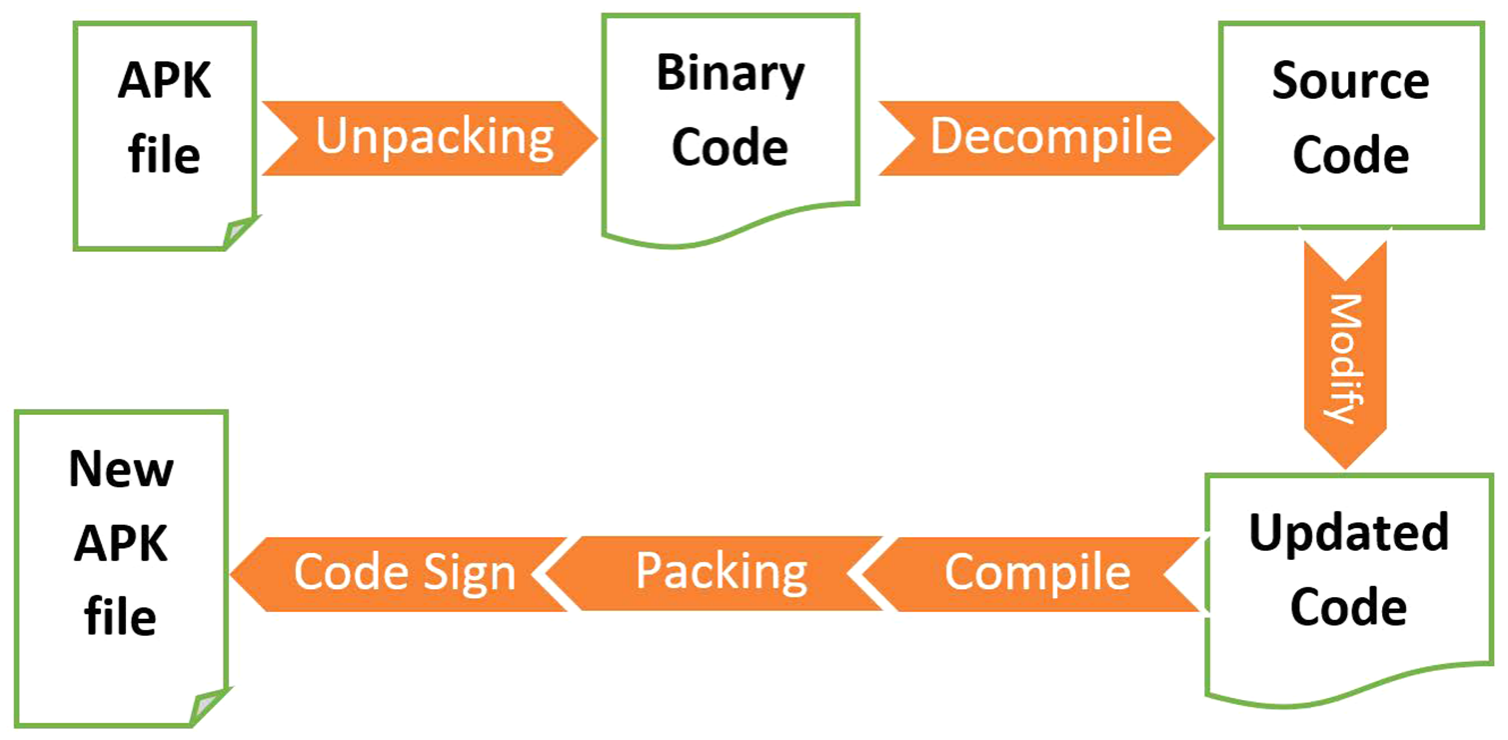
App repacking sequence.

- ***RePacking* (RP):** Since most anti-malware tools rely on signatures, a simple obfuscation mechanism is to decompress the APK file, add junk code or malicious code, and then recompresses the APK file (Hammad, Garcia & Malek, 2018) (as shown in Fig. 2).
- ***Junk Code Insertion* (JCI):** This technique does not change the flow or logic of the program. A random amount of code is inserted in each method executed, however, it does not affect the rest of the program (Hammad, Garcia & Malek, 2018). The goal of this technique is to change essential code signatures, *e.g*., by increasing the size of the App.
- ***Try-Catch* (TC):** This technique always executes the catch block by inserting an exception into the try block (Preda & Maggi, 2017). This obfuscated example creates the illusion that the catch block is rarely executed when an error occurs in a try block. However, the error is always present in the try block, and the catch block is always executed.
- ***String Encryption* (SE):** The strings in the code can be used as signatures (Graux, Lalande & Tong, 2019) to identify the malicious behavior of the application. This technique is used to encrypt strings. Encrypting the strings makes signature-based detection more difficult. For example, a **plaintext** is stated as “*defend the east wall of the castle*”, which can be encoded as the **ciphertext** as “*efgfoe uif fbtu xbmm pg uif dbtumf*”.
- ***Call Indirection* (CI):** Some anti-malware tools use the structure of method call graphs to generate signatures (Hammad, Garcia & Malek, 2018). The original method call can be modified by inserting a randomly generated proxy method before the original method call. Each method call is redirected to the proxy method, and the proxy method forwards the call to the original method with the same parameters.
- ***Opaque Predicate* (OP):** This obfuscation uses conditional expressions to ensure that one branch is always gets executed (Chua & Balachandran, 2018). This technique can be combined with the junk code insertion obfuscation technique. An opaque predicate statement is inserted between the original code and always set to execute one branch.
- ***Code Reordering* (CR):** This technique aims to randomly change the order of instructions however it preserves the original execution order of the instruction by adding *goto* the instruction (Hammad, Garcia & Malek, 2018). The signature generated by this technique is significantly different from the original signatures because of the order of the instructions.
- ***Identifier Renaming* (IR):** This obfuscation mechanism replaces each class, method, and field name with random strings (Hammad, Garcia & Malek, 2018). The identifier is renamed by the random encrypted string, which can be generated by various algorithms.
- ***Data Encoding* (DE):** This technique is used to encode strings and arrays (Hammad, Garcia & Malek, 2018). The strings and arrays in the code can be used as a signature to identify the malicious behavior of the application. By encrypting strings and arrays, signature-based detection is no longer as effective.
- ***Members Reordering* (MR):** This technique aims to change the order of variables or methods in a program (Hammad, Garcia & Malek, 2018). If the anti-malware product depends on a sequence of variables and methods, it can be easily bypassed using this technique.
- ***Package Renaming* (PR):** The application package name is specified in the application manifest file (Preda & Maggi, 2017). Packages are defined by using slashes instead of the dot-like directory structure, which is a unique identifier for each application.
- ***Reflection* (Re):** This technique aims to hide the methods and fields that the code calls at runtime (Graux, Lalande & Tong, 2019). The original method is called another method to redirect the execution flow. Each method call is redirected to a new method, and that method redirects the calls to the original method with the same parameters. The calling mechanism is known as reflection.
- ***Disassembling and Reassembling* (DRe):** This technique simply disassembles and reassembles the application using the APK tool (Hammad, Garcia & Malek, 2018). Disassembling and reassembling the app reorders the order of the elements in the classes.dex file.
- ***Android Manifest Transformation* (AMT):** Each Android application contains a configuration file called *androidmanfest.xml* (Hammad, Garcia & Malek, 2018). Various permissions are defined in it. We can change the permissions or add new ones. Some of the permissions include *android.permission. ACCESS _BLUETOOTH_SHARE*, which is used for Bluetooth sharing.
- ***Resource Renaming* (RR):** This technique parses the name from the XML file extracted from the APK file and renames the resource name to an arbitrary encrypted string (Preda & Maggi, 2017). The resources are available in the res folder in the Android application, which is changed after the application is extracted.
- ***Method Overloading* (MO):** This technique uses the overloading feature of the JAVA language to assign the same name to different methods with different additional arguments (Chua & Balachandran, 2018). If the method name already exists, this technique creates a new method with the same name and different arguments.
- ***Re-Order Loop* (ROL):** This technique (Graux, Lalande & Tong, 2019) is used to change the control flow of any application. A loop can be executed in reverse order by changing conditions and variables in a loop. The simple order of the control flow of any application is changed with this technique.
- ***Unconditional Jump Insertion* (UJI):** This technique (Faruki et al., 2016) is used to change the control flow structure of any application. Goto-statements introduce unconditional jumps. Forward and backward jumps are introduced, but the functionality of the program remains the same.
- ***Debug Removal* (DebR):** This technique removes all debug information (Preda & Maggi, 2017) that is by default enabled by the developers. By allowing the debugger, you help the developer find the location of the error log information. This technique removes all directories where the error logs are stored. By eliminating the log directories, the developer cannot find out where exactly the errors occurred.
- ***Re-Aligning* (RA):** This technique (Preda & Maggi, 2017) realigns all uncompressed data, including images and miscellaneous files in the APK file. This realignment changes the signature of the APK file. This technique can easily bypass the anti-malware tools that detect the malicious app based on its signature.

**Table 3:** Obfuscation techniques and impact on app code and manifest file.

Obfuscation technique	Code changes	Manifest changes	Obfuscation technique	Code changes	Manifest changes
Repackaging	✓	✗	Junk Code Insertion	✓	✗
Try-catch	✓	✗	String Encryption	✓	✗
Call Indirection	✓	✗	Opaque Predicate	✓	✗
Code Reordering	✓	✗	Identifier Renaming	✓	✗
Data Encoding	✓	✗	Members Reordering	✓	✗
Package Renaming	✗	✓	Reflection	✓	✗
Disassembling, Reassembling	✓	✗	Manifest transformation	✗	✓
Resource Renaming	✓	✗	Method Overloading	✓	✗
Re-order Loop	✓	✗	Unconditional Jump	✓	✗
Debug Removal	✗	✓	Re-aligning	✓	✗

## Evaluating Anti-Malware Tools

This section explains the proposed research methodology for evaluating anti-malware tools. The main objective of this work is to provide a comprehensive study for the evaluation of anti-malware tools. In this work, 20 major obfuscation techniques are discussed and applied to malware samples in isolation, inter-category-wise, and inter-category. The obfuscated malware files are then analyzed with known anti-malware tools to evaluate the effectiveness of detection. To manage the large number of combinations for the 20 obfuscation techniques, we divide these techniques into several categories. Then, we deploy these obfuscation combinations in the hybridized form by using inter-category-wise and inter-category hybridization combinations. In code obfuscation, the application code is modified. However, the functionality of the code is preserved. Obfuscation can be used to hide malicious code in benign code, making it difficult to determine whether the code could perform the malicious activity or not. Before applying obfuscation, we need to extract the source code of the application. After extracting the application code, the obfuscation techniques with possible combinations are applied to the APK file. This obfuscated APK may contain malicious code or other malicious activities. Now, this obfuscated APK is analyzed using the anti-malware tools.

**Figure 3:**
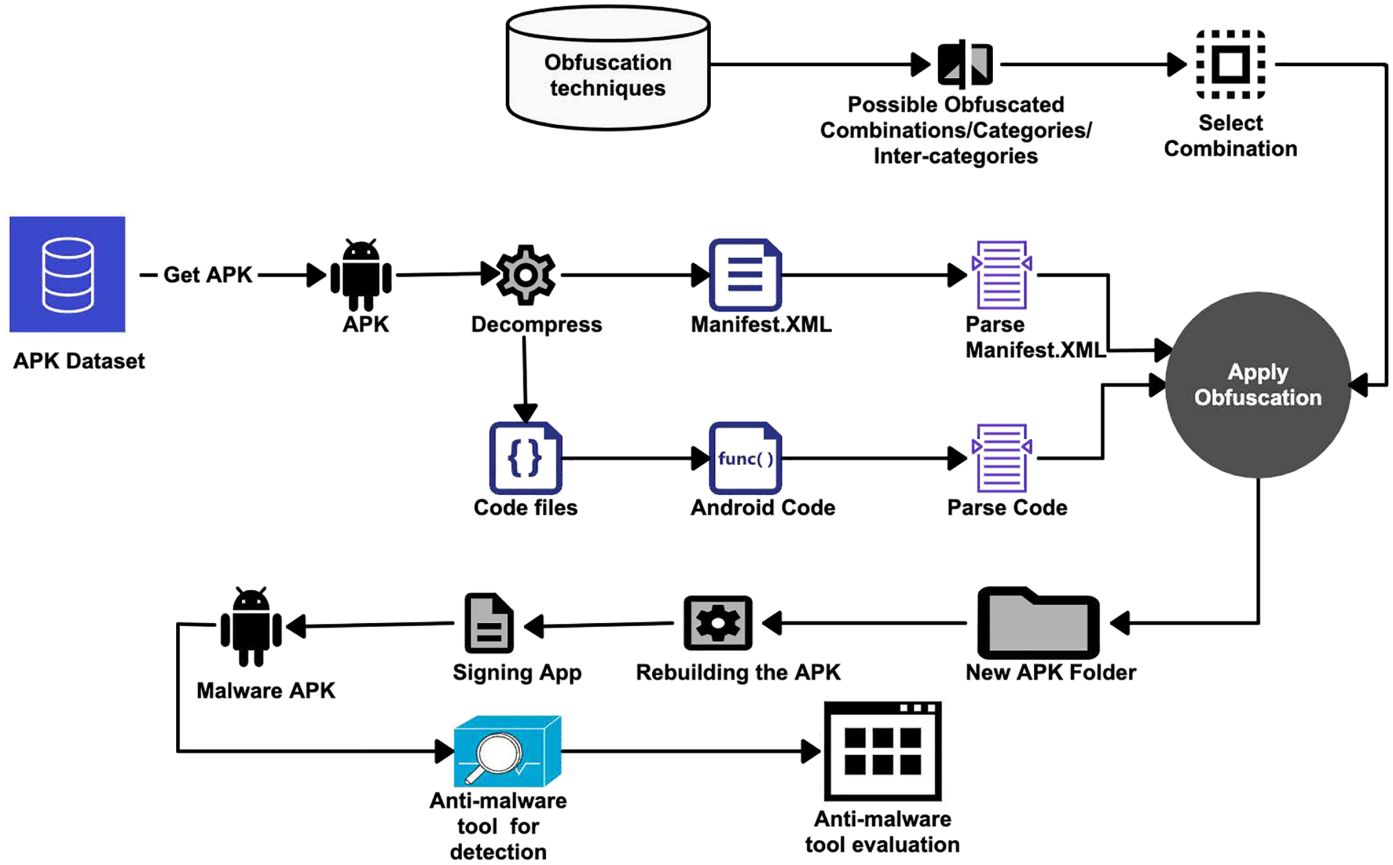
Anti-malware tools evaluation methodology.

As shown in Fig. 3, the APK dataset is used to obtain the APK files. After obtaining an APK from the dataset, the first phase is to extract the resource files (*i.e*., resources, manifests, and other code files) from the APK using APKTools (Apktool, 2022) or another equivalent software program. The extracted *manifest.xml* contains all configurations for the application. All permissions for using the application are specified in the manifest file. The user must grant these permissions at the time of installing the Android application. In general, a program cannot access application components and other resources without certain permissions. We have also extracted the source code files using APKTool. The extracted source code is in the form of Java or Smali code. We use the Smali code of an application to make obfuscation-related changes. Before applying any code modification, it is necessary to check whether it is possible to apply a particular obfuscation in the code or not. Also, the program execution flow is closely examined to ensure that the obfuscation does not change the semantics or meaning of the program. Considering the 20 obfuscation techniques used and their further classification into categories or classes (as in Table 4), the possible combinations are formed. The obfuscation techniques are applied in isolation, category by category, and inter-categories.

**Table 4:** Category wise distribution of obfuscation techniques.

Category	Obfuscation techniques
Android Specific (AS)	• Repackaging
	• Disassembling and Reassembling
	• Android Manifest transformation
	• Re-aligning
Simple Control-flow Modifications (SCF)	• Junk Code Insertion
	• Debug Removal
	• Try-catch
	• Members reordering
	• Re-order Loop
Advanced Control-flow Modifications (ACF)	• Call Indirection
	• Code Reordering
	• Reflection
	• Opaque Predicate
	• Method Overloading
	• Unconditional jump insertion
Renaming (RN)	• Package Renaming
	• Identifier Renaming
	• Resource Renaming
Encryption (EN)	• String Encryption
	• Data Encoding

Furthermore, the obfuscated APK folder is made available to the APKTool so that the application can be rebuilt. After rebuilding, the APK is signed with the developer’s unique key. The Jarsigner tool is used for signing. The signed application looks like a non-obfuscated application sample, which can hide malicious activities. For analysis, the obfuscated malware is provided to the anti-malware tools to see whether these tools can detect the malware or not.

### Obfuscation techniques

In Table 4, 20 different obfuscation techniques are mentioned, which can be divided into five categories. The Android-specific obfuscation techniques leave the bytecodes unchanged while applying the obfuscations. The simple control flow modification-related obfuscation techniques are based on the mechanisms that potentially change or add new code in an application; however, simple control flow modification techniques deal with the simpler code. The advanced control flow modification techniques mostly involve the obfuscation approaches that use more complicated and advanced steps to modify the application code. However, both simple and advanced control flow modifications require changes to the application code while keeping the semantics of the code the same. Renaming-related obfuscation techniques include all techniques that use renaming mechanisms for application resources such as folders, code, and so on. For example, the package renaming technique does not change anything but renames the application packages in the application folder. Some of the renaming techniques can also change the application code, such as renaming identifiers. Encryption-based mechanisms include any technique that encrypts the code, data, strings, or application resources such as folders.

## Experimental Evaluation

This section presents the evaluation of the top 10 commercially available prominent Android malware detectors. The malware samples are taken from the Drebin dataset (Arp et al., 2014). The experimental evaluation presented in this section shows the potential impact of obfuscation mechanisms on the detection rate of these anti-malware tools.

### Malware dataset

Drebin (Arp et al., 2014) is a comprehensive dataset based on a large number of Android malware, *i.e*., 5,560 malicious APK files belonging to 179 different malware families. We selected some random malware samples and used them as base APKs to create 700 different obfuscated samples for evaluation, which can be observed in Table 5.

**Table 5:** Evaluated anti-malware tools.

Anti-malware tool, reference	Current version	Total downloads	Ratings	Offered by
Avast, (Avast, 2022)	6.38.2	100M+	4.7	Avast Software
AVG Mobile, (AVG Technologies, 2022)	6.38.4	100M+	4.7	AVG Mobile
Kaspersky, (Kaspersky, 2022)	Varies with device	50M+	4.8	Kaspersky Lab
McAfee, (McAfee, 2022)	5.13.0.136	50M+	4.5	McAfee LLC
Avira, (Avira, 2022)	7.7.1	10M+	4.6	AVIRA
Dr. Web, (Web, 2022)	12.6.9	10M+	4.6	Doctor Web, Ltd
ESET Mobile Security, (ESET, 2022)	6.3.41.0	10M+	4.8	ESET
Malwarebytes Security, (Malwarebytes, 2021)	3.7.5.8	10M+	4.6	Malwarebytes
Bitdefender, (Bitdefender, 2022)	3.133.939	5M+	4.7	Bitdefender
Sophos, (Sophos, 2022)	9.6.3415	1M+	4.3	Sophos Limited

We have selected the 10 popular and prominent anti-malware tools for evaluation. These anti-malware tools were selected based on the number of downloads and ratings in the Play store. The choosing criteria for these anti-malware tools are a minimum of 4.3 ratings with a large number of downloads (minimum of 1 million downloads). Table 5 shows the anti-malware tools considered in this study including the metadata such as number of downloads, current version, and star rating against, *etc*. We use the VirusTotal (virusTotal, 2012) platform, which helps us scan the obfuscated samples with one of these tools.

### Experimental setup

To reverse engineer an Android APK, we use Apktool (Apktool, 2022), which generates Smali code files. We apply the intended obfuscation techniques to the Smali code obtained from these APKs by using the Visual Studio tool (Visual Studio Code, 2016) (a lightweight source code editor). The details of the machines used in the experiments can be found in Table 6.

**Table 6:** Machine specifications.

CPU	Intel core I5 2.5 GHz
Installed RAM	8 GB
Operation System	Windows 10 Pro
Reverse engineering Tool	Apktool v2.5.0
Tool for Smali code edit	Visual studio code v1.56.2

### Results

Twenty different malware sample APKs are obfuscated and a total of 700 variants are developed for evaluation. These twenty malware samples are selected from the Drebin dataset (Arp et al., 2014). These malware samples are detected initially as malware by all anti-malware tools available in this study. We create 400 variants using single obfuscation techniques (*i.e*., in isolation). One hundred malware variants are created using obfuscation techniques by category, while 200 malware variants are created using inter-category combinations. The evaluation results for each anti-malware tool are presented in three diagrams. The first chart shows how effective the obfuscation techniques are when used in isolation. The second chart shows the detection performance when inter-category obfuscation. Details on inter-category obfuscation can be found in Table 5. The designed model is applied to the 100 malware samples. The third chart shows the detection performance of the anti-malware tools when tested against the obfuscated samples based on inter-category obfuscation. Below are the detailed evaluation results of each anti-malware tool.

#### Avira

**Figure 4:**
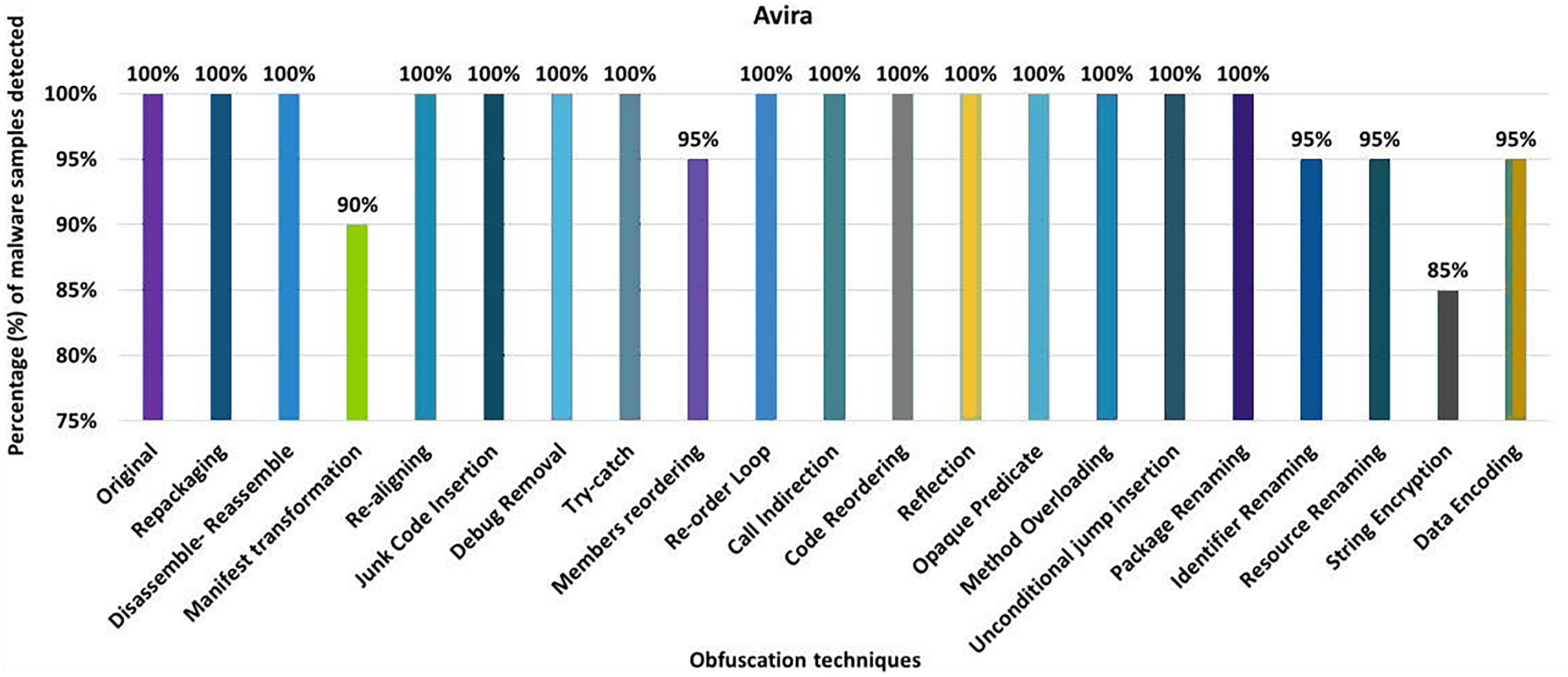
Avira: isolated obfuscation evaluation.

**Figure 5:**
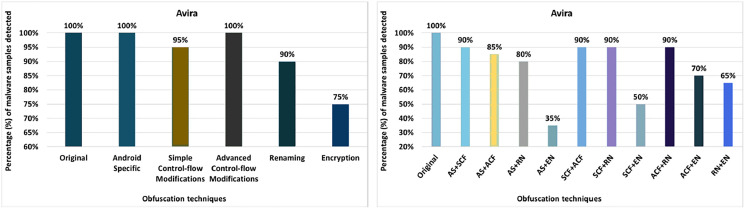
Avira: category-wise and inter-category-wise results. (A) Avira: category-wise obfuscation results. (B) Avira: inter-category-wise obfuscation results.

The detection rate of the Avira anti-malware tool shows that 100% of the original malware samples were detected as malicious APKs. The evaluation results for the isolated application of obfuscation techniques show that Avira’s detection rate drops to 85% after applying the obfuscation associated with string encryption. In its original form, Avira was able to detect 100% of files as malicious. However, after creating variants of malicious APKs by applying string encryption, Avira’s performance drops to 85%, as seen in Fig. 4. The results show that Android manifest conversion, member reordering, identifier renaming, resource renaming, and data encryption also degraded the detection rate a bit. When obfuscation techniques are applied in the category-by-category configuration, Avira’s detection rate drops to 75% (for example, for the encryption category), as shown in Fig. 5A. Simple control flow changes and category renaming also have little effect on the detection rate of the Avira anti-malware tool, as shown in Fig. 5A.

When obfuscation techniques are applied with inter-category configurations, Avira’s detection rate drops to 35% with a combination of Android-specific and encryption (as shown in Fig. 5B). These experiments highlight the fact that the combinations between the categories for obfuscation are inherently stealthier and significantly reduce the detection capability.

In experiments, the minimum detection rate decreases from 100% to 85% when the obfuscation techniques are applied in isolation, from 100% to 75% when they are applied category-wise, and from 100% to 35% when the obfuscation techniques are combined inter categories. The results show that the detection rate drops significantly when the obfuscation techniques are combined with inter-categories.

#### Avast

**Figure 6:**
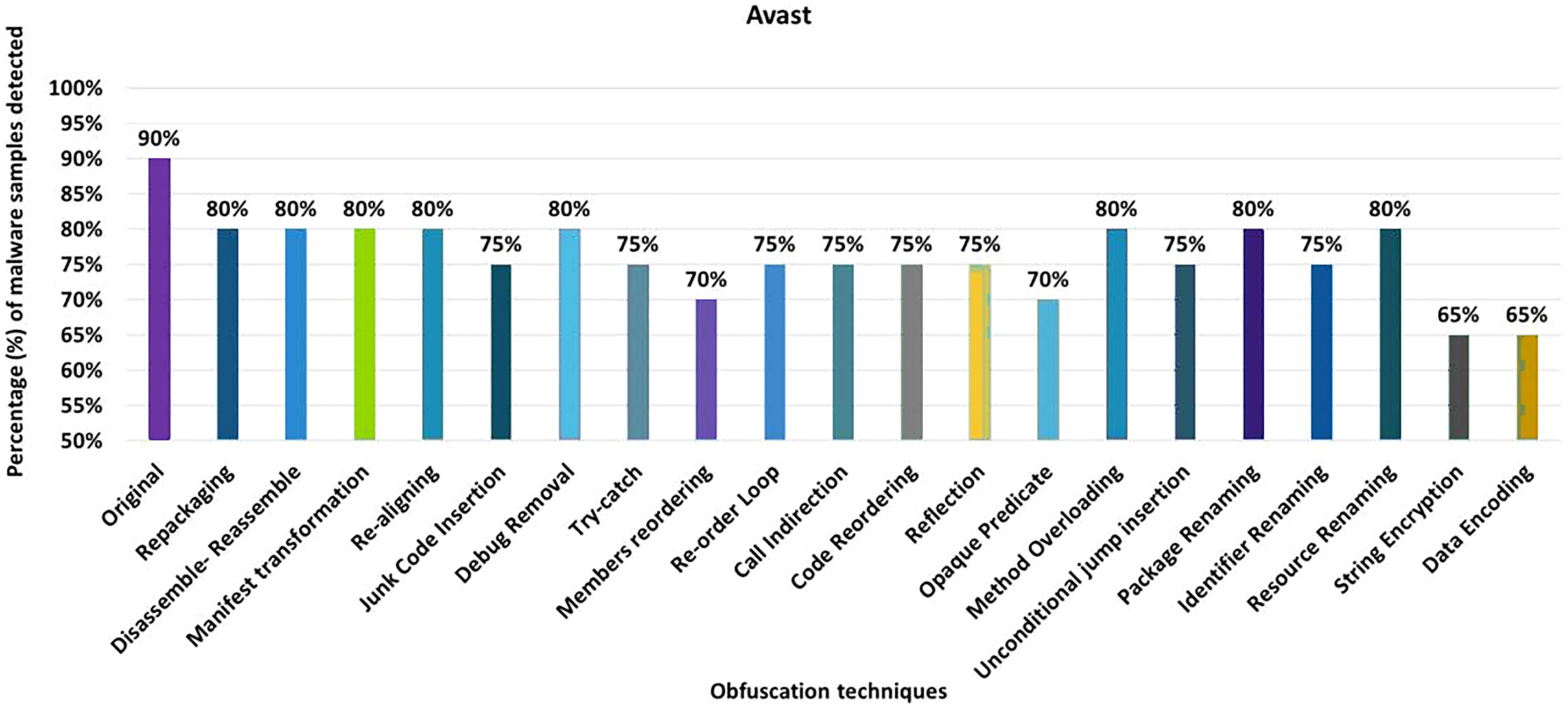
Avast: isolated way obfuscation results.

The detection rate of the Avast anti-malware tool shows that 90% of the original malware samples (without any obfuscation) were detected as malicious applications. However, after applying 20 different obfuscation techniques (*i.e*., in isolation), the detection rate dropped to 65%, which can be observed in Fig. 6. The notable obfuscation techniques that caused a drop in detection rate are string encryption and data ending.

**Figure 7:**
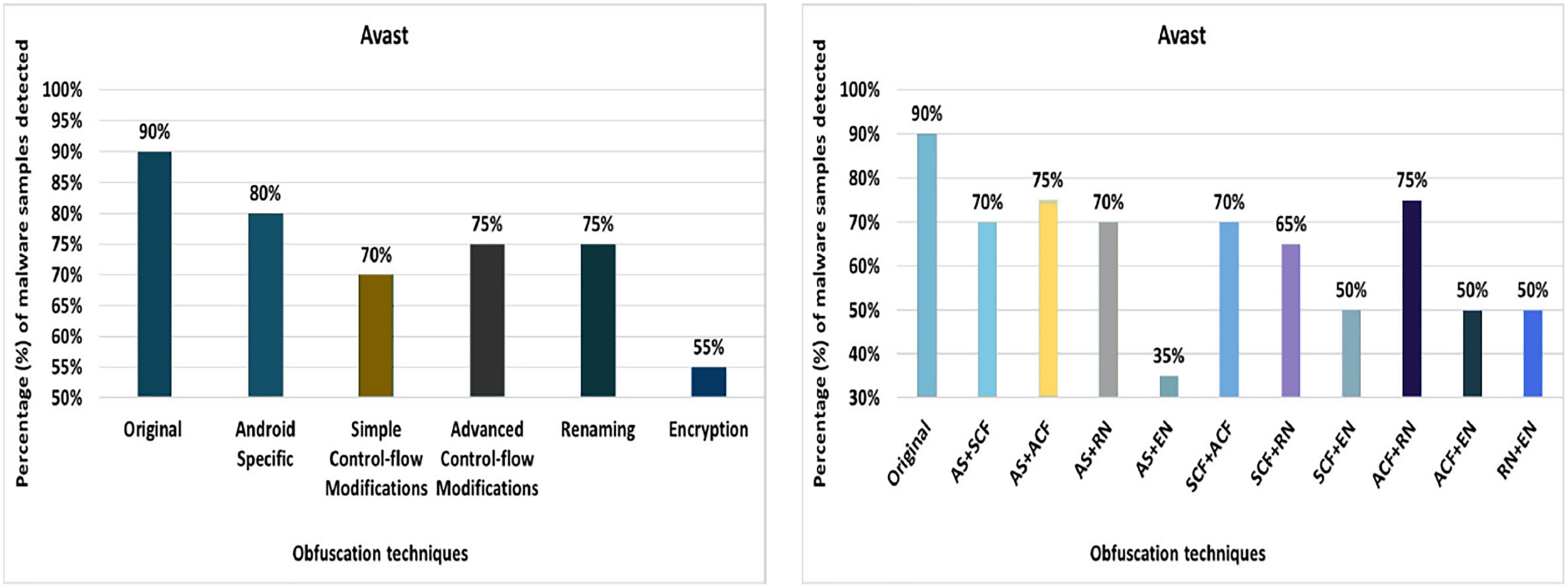
Avast: category-wise and inter-category-wise results. (A) Avast: category-wise obfuscation results. (B) Avast: inter-category-wise obfuscation results.

When obfuscation techniques are applied on a category-by-category basis, Avast’s detection rate drops from 90% to 55%, especially in the case of the encryption category, as shown in Fig. 7A. The evaluation results for applying obfuscation techniques in a category show a significant drop in the detection rate, *i.e*., 55% for the Encryption category (as shown in Fig. 7A).

When obfuscation techniques are applied inter categories, Avast’s detection rate drops further to 35% when Android-specific and encryption categories are combined, which can be seen in Fig. 7B. The most significant drop was caused by the combination of Android-specific and encryption categories, while the other inter-category combinations cause a notable drop in the detection rate.

#### AVG mobile

**Figure 8:**
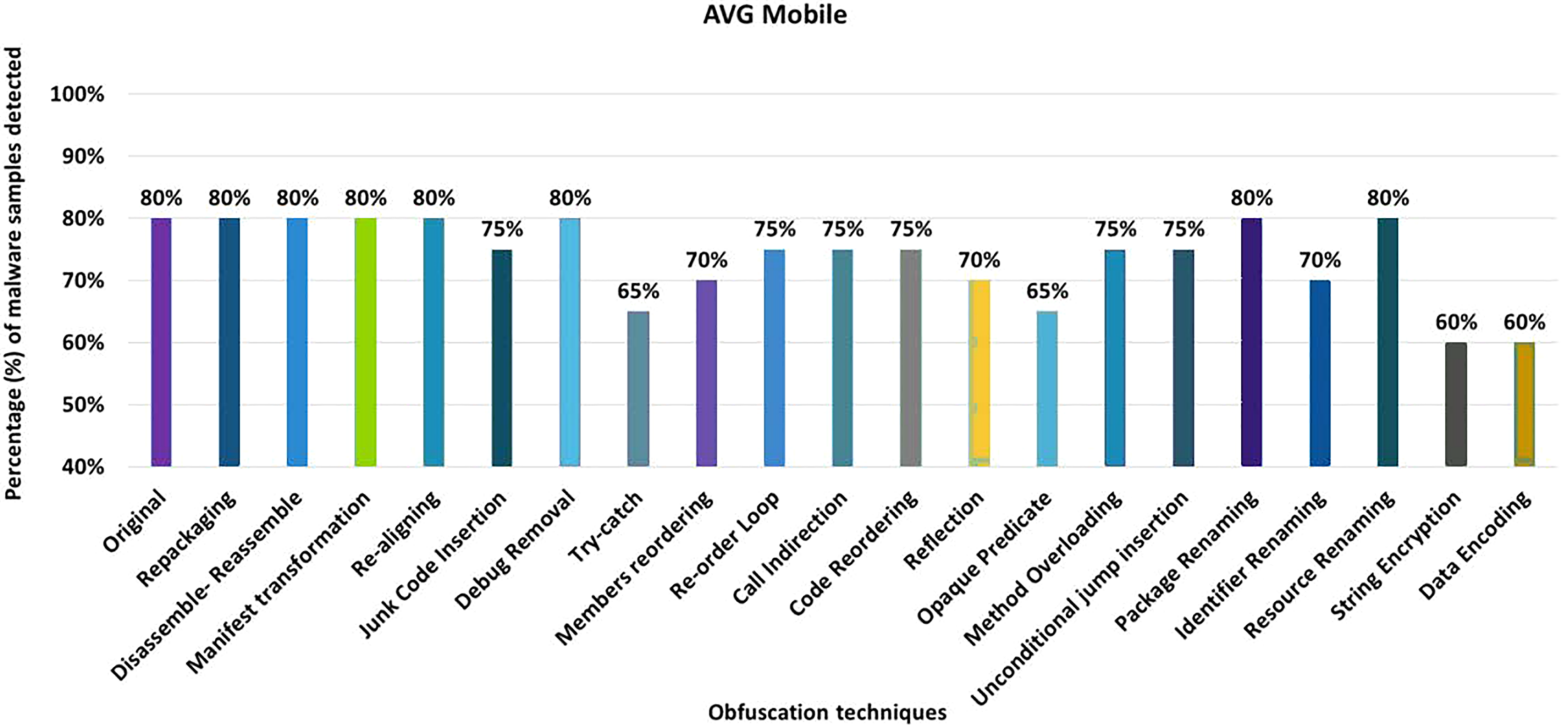
AVG Mobile: isolated way obfuscation results.

AVG Mobile anti-malware tool detects 80% of the original malware samples as malicious applications. The evaluation results when obfuscation techniques are applied in isolation show that the detection rate of AVG Mobile drops to 60% (as shown in Fig. 8). The decrease in detection rate is mainly due to string encryption, data encryption, application of additional try-catch blocks, opaque predicates, and code reordering mechanisms.

**Figure 9:**
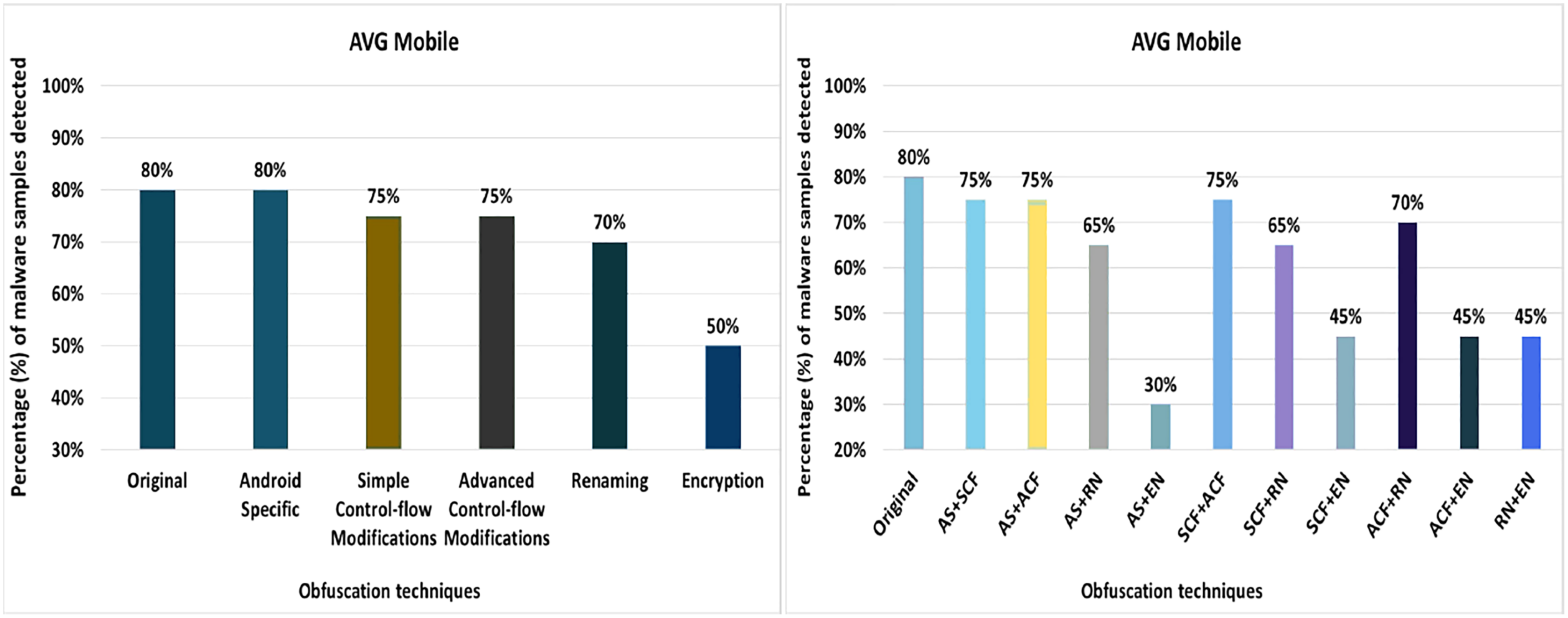
AVG Mobile: category-wise and inter-category-wise results. (A) AVG Mobile: category-wise obfuscation results. (B) AVG Mobile: inter-category-wise results.

When obfuscation techniques are applied on a category-by-category basis, the detection rate of AVG Mobile decreases from 80% to 50%, as shown in Fig. 9A. The results show that the categories of simple-control-flow, advanced-control-flow and renaming have a significant impact on the detection rate of the anti-malware tool AVG Mobile, that can be seen in Fig. 9A.

When obfuscation techniques are applied inter categories, the detection rate of AVG Mobile drops to 30% with a combination of Android-specific and encryption techniques that can be seen in Fig. 9B. The effects of the other inter-category obfuscation techniques are also notable, as can be seen in Fig. 9B.

#### Bitdefender

**Figure 10:**
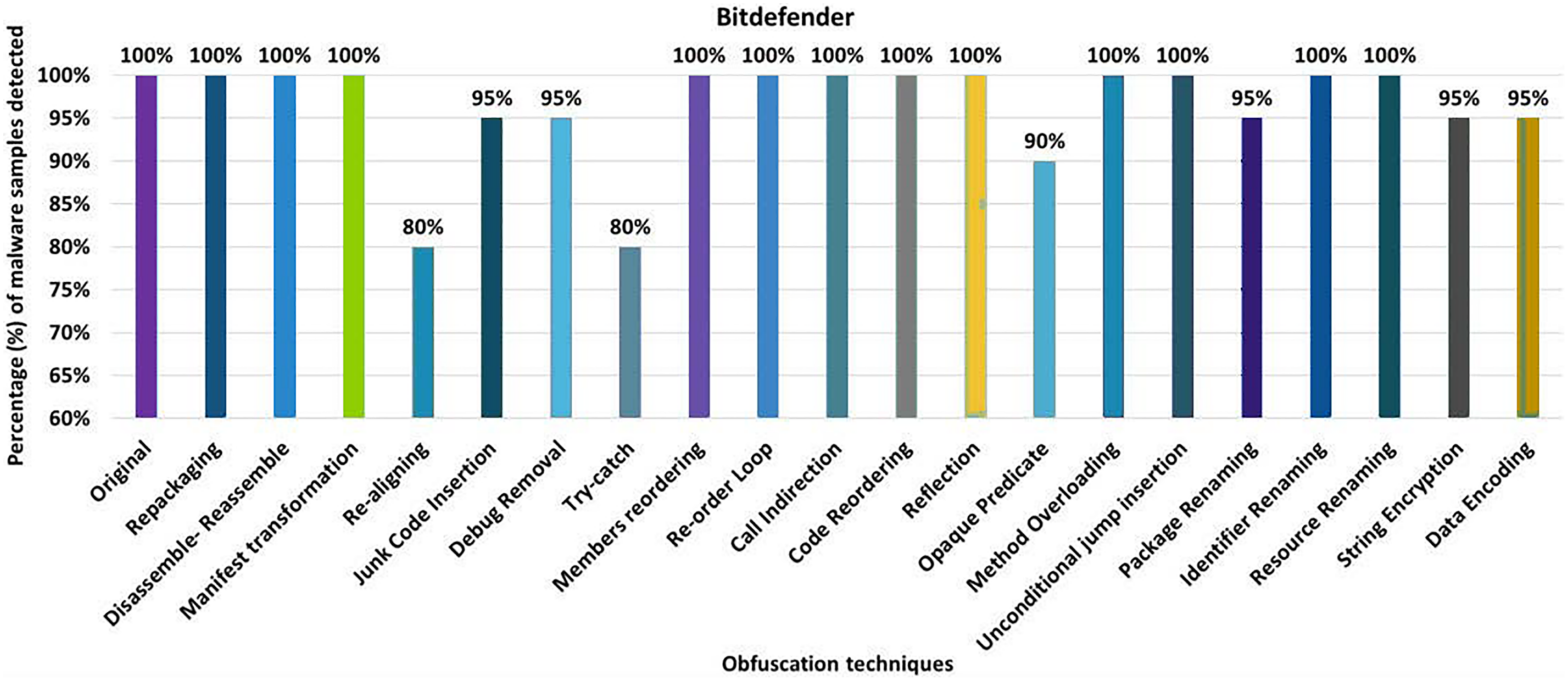
Bitdefender: isolated way obfuscation results.

This work shows that 100% of the original malware samples were detected as malicious by Bitdefender anti-malware tool. After applying 20 different obfuscation techniques, one by one, the results dropped to a detection rate of 80%. The evaluation results for the isolated application of obfuscation techniques show that Bitdefender’s detection rate dropped to 80% after applying re-aligning or try-catch, as shown in Fig. 10. The results show that opaque-predicate, package-renaming, string-encryption and data-encryption also affect the detection rate of the Bitdefender anti-malware tool.

**Figure 11:**
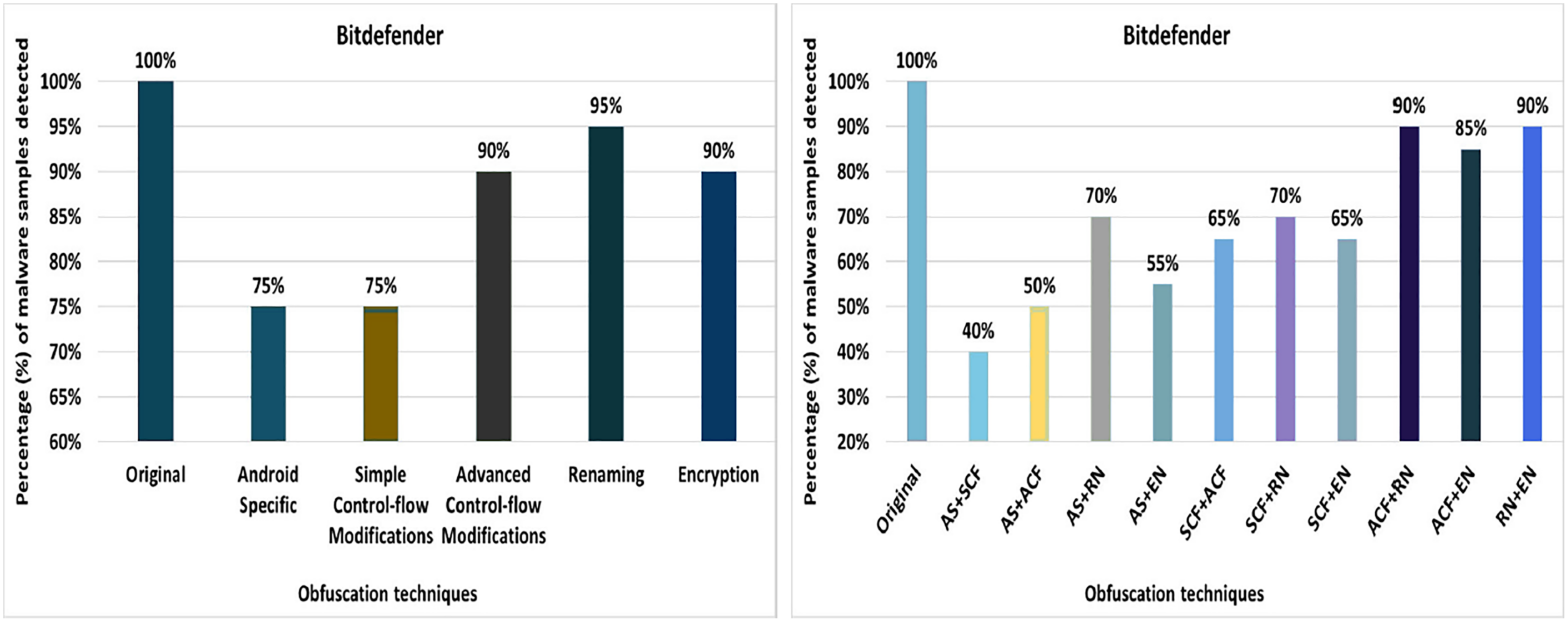
Bitdefender: category-wise and inter-category-wise results. (A) Bitdefender: category-wise obfuscation results. (B) Bitdefender: inter-category-wise obfuscation results.

When the obfuscation techniques are applied category-wise, the detection rate of the Bitdefender anti-malware tool in the Android-specific and simple control flow modifications category drops from 100% to 75% that can be found in Fig. 11A. These results highlight the fact that all applied category-specific obfuscations led to a significant decrease in the detection rate.

When obfuscation techniques are applied to inter-categories, Bitdefender’s detection rate drops to 40% (for the inter-category combination of Android-specific and simple control flow modifications), as shown in Fig. 11B. The results also show that the other inter-category combinations also have a remarkable impact on the detection rate of the Bitdefender anti-malware tool.

#### Dr. Web

**Figure 12:**
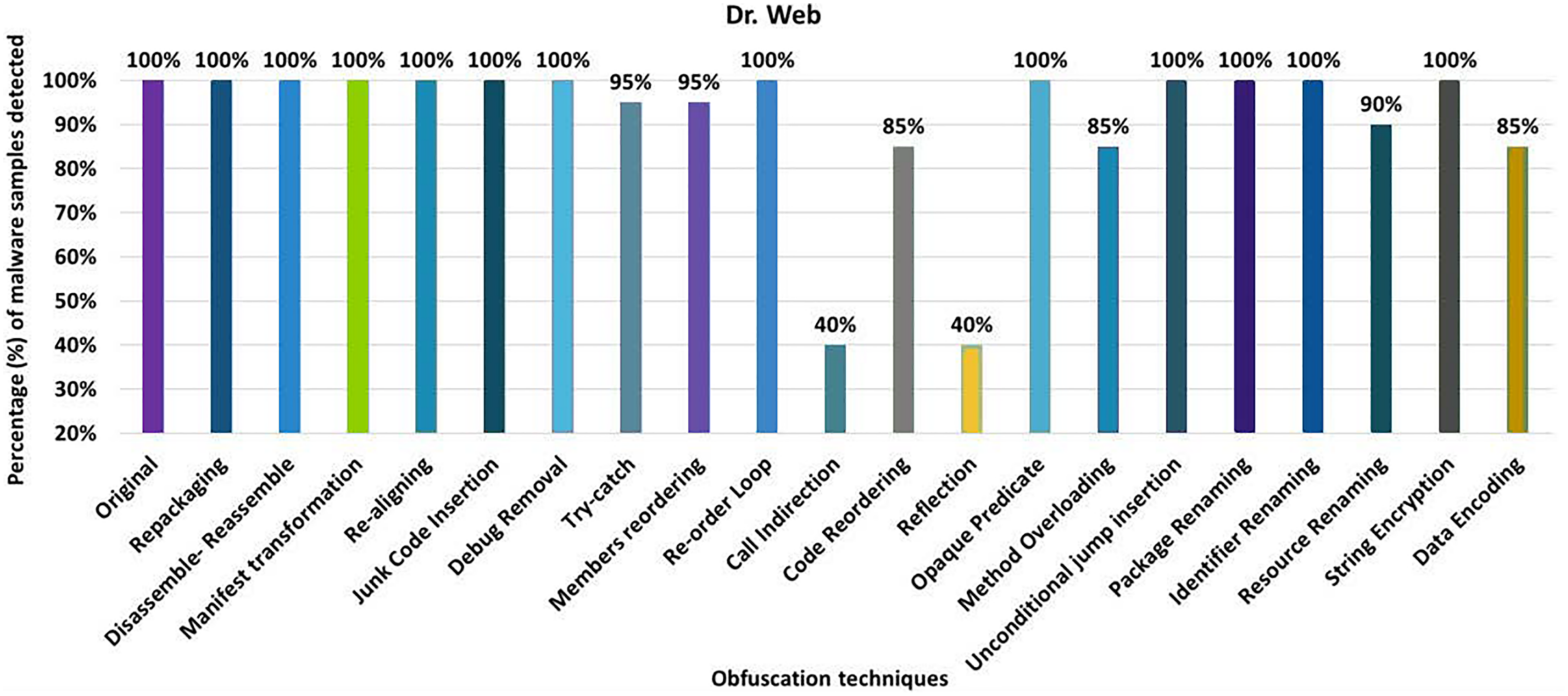
Dr. Web: isolated way obfuscation results.

Dr. Web’s detection rate for the original malware was 100%, as seen in Fig. 12. After applying 20 different obfuscation techniques, one after another, the detection rate dropped to 40% (especially after applying call indirection or reflection obfuscations). Moreover, the results show that the code-reordering, resource-renaming, and data encoding have a significant impact on the detection rate of the Dr. Web anti-malware tool.

**Figure 13:**
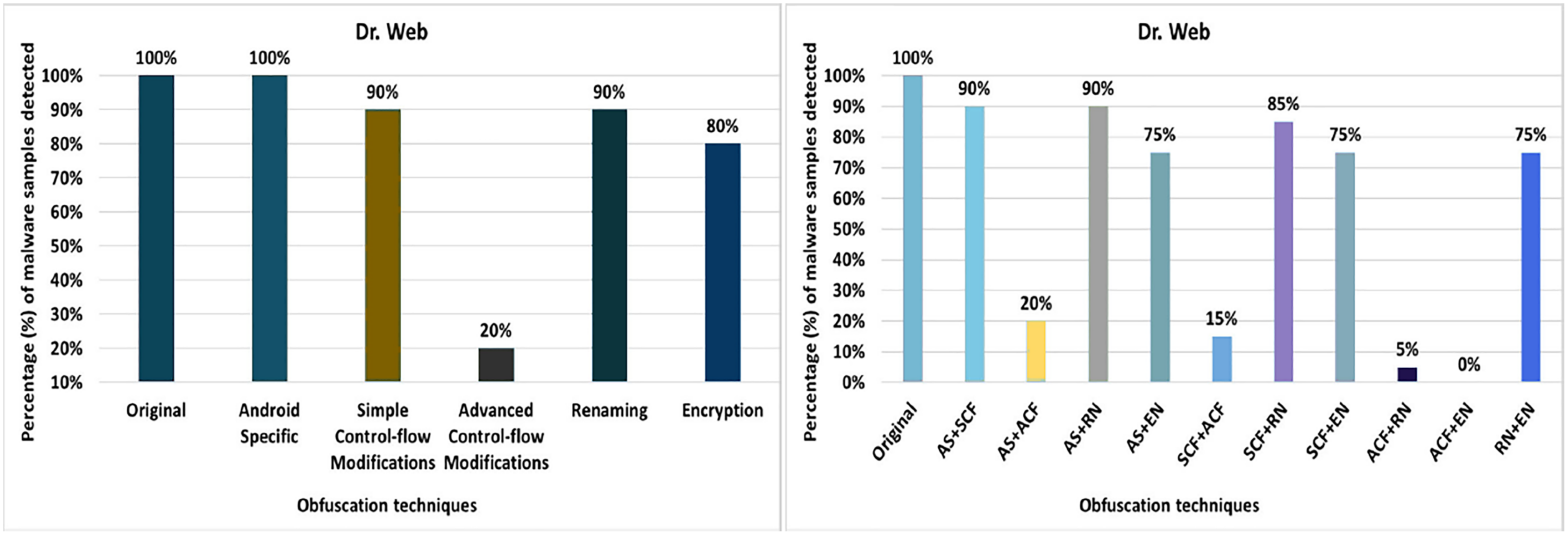
Dr. Web: category-wise and inter-category-wise results. (A) Dr. Web: category-wise obfuscation results. (B) Dr. Web: inter-category-wise obfuscation results.

When obfuscation techniques are applied category-wise, the detection rate drops significantly from 100% to 20%. All category-wise combinations have a remarkable effect on the detection rate; however, the advanced control-flow modifications has a high impact, as shown in Fig. 13A. For the inter-category wise combination, the detection rate dropped drastically to 0% for the combinations of advanced control-flow modifications and encryption, as shown in Fig. 13B. In addition, the combination of advanced control-flow modifications and renaming had a significant impact on the detection rate (*i.e*., it dropped to 5%). The other inter-category combinations between categories also had a large impact on the detection rate, as shown in Fig. 13B.

#### ESET mobile security

**Figure 14:**
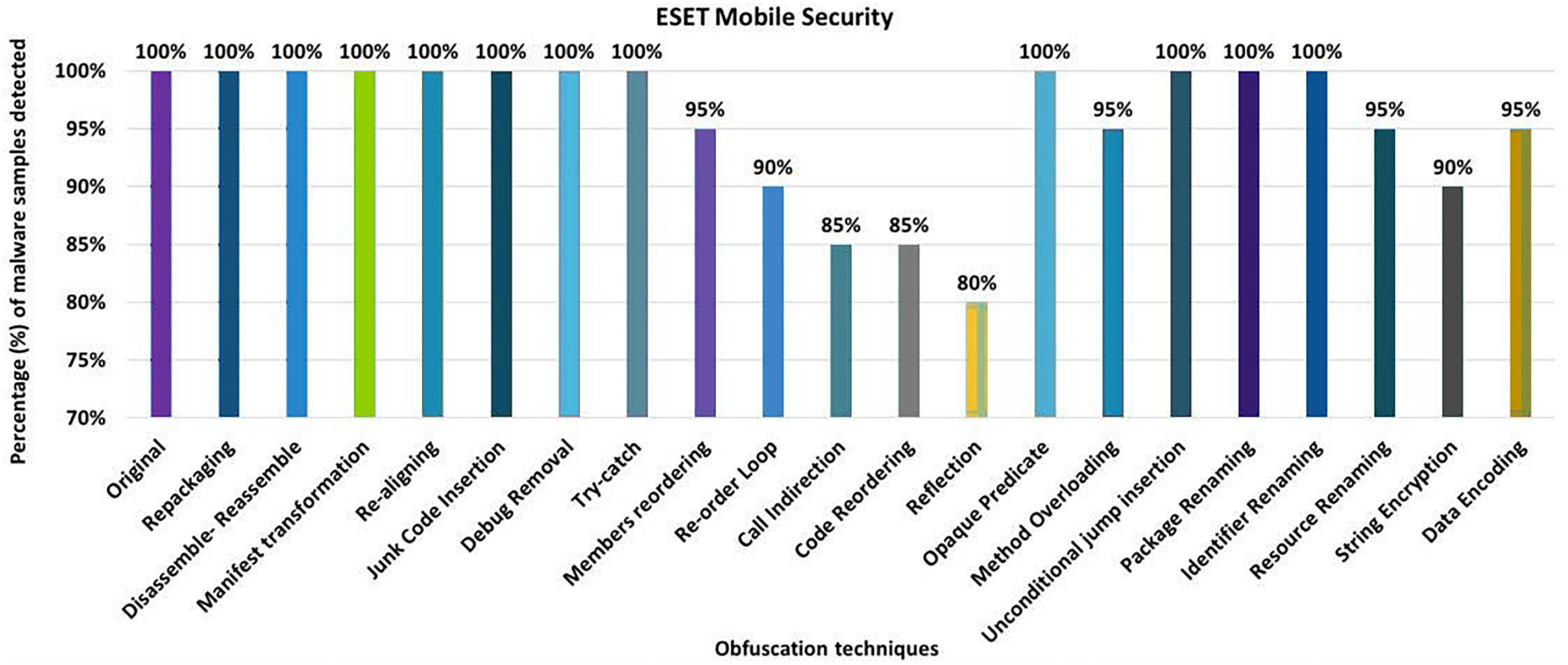
ESET Mobile Security: isolated way obfuscation results.

The ESET Mobile Security tool detected 100% of the original malware samples as malicious. However, when it was equipped with the 20 different obfuscation techniques (one at a time), the detection rate dropped to 80%, as shown in Fig. 14. The results show that obfuscations such as Members Re-Ordering, Re-Order-Loop, Call Indirection, Code Re-Ordering, Identifier Renaming, Resource Renaming, and Data Encoding also have a notable impact on the detection rate.

**Figure 15:**
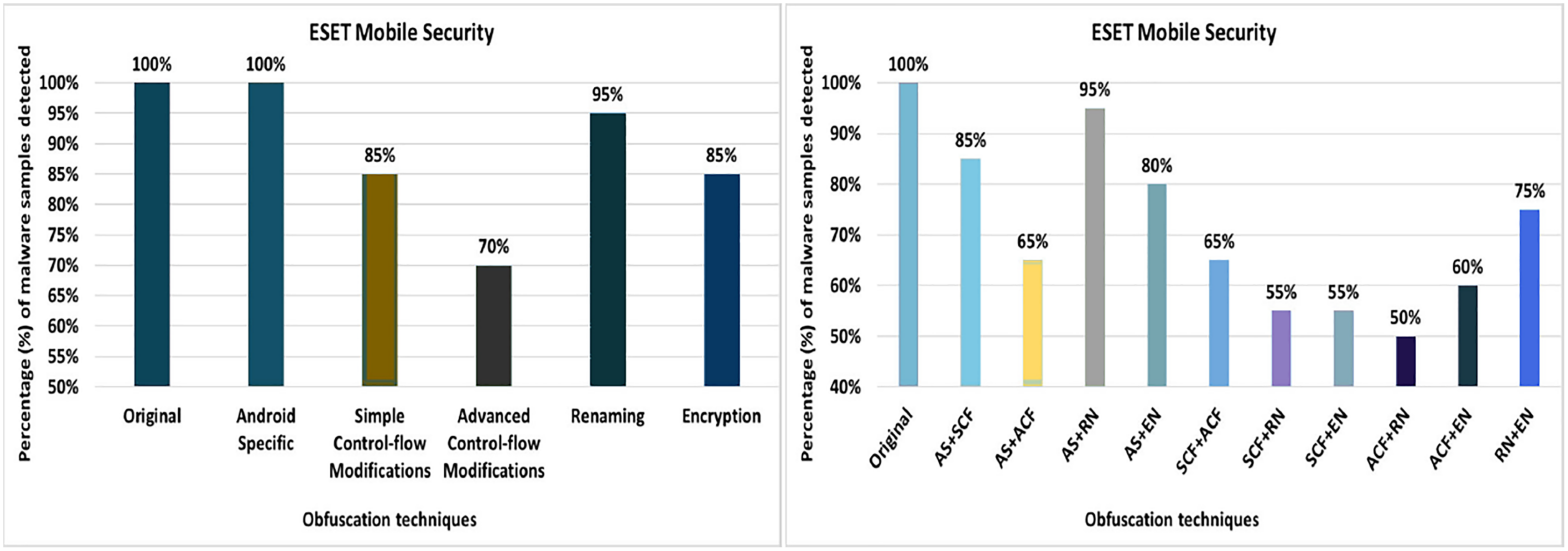
ESET Mobile Security: category-wise and inter-category-wise results. (A) ESET Mobile Security: category-wise obfuscation results. (B) ESET Mobile Security: inter-category-wise obfuscation results.

When obfuscation techniques are applied category by category, the detection rate of ESET Mobile Security drops to 70%. The maximum drop in detection rate was observed when the Advanced control-flow modifications obfuscation category was applied (as shown in Fig. 15A). The other category-related obfuscations also have a notable impact on the detection rate.

When obfuscation techniques are applied inter categories, ESET’s detection rate drops further to 50% (*i.e*., for the combination of advanced control-flow modifications and *renaming*), as shown in Fig. 15B. The experiments also found that all combinations of intermediate categories also have a significant impact on the detection rate of the ESET anti-malware tool.

#### Kaspersky

**Figure 16:**
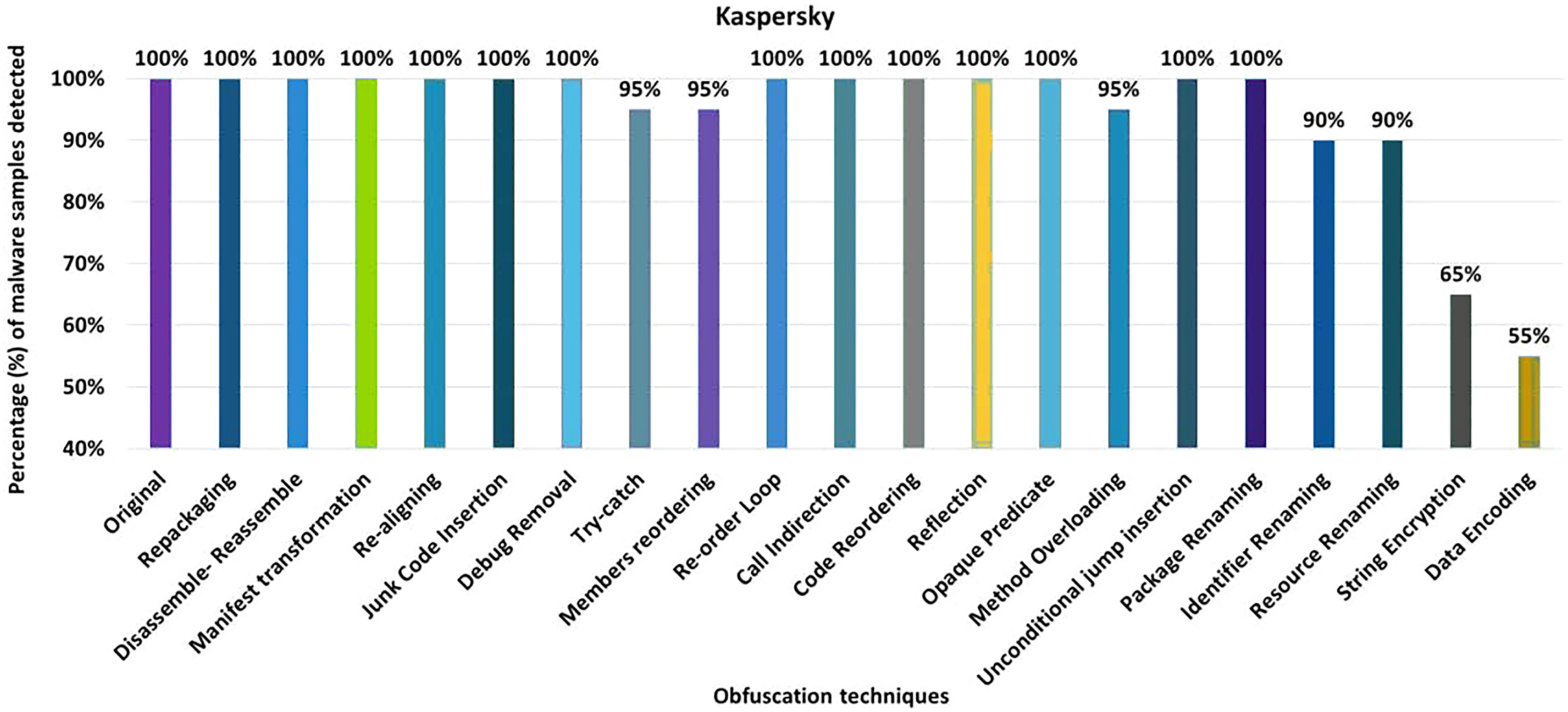
Kaspersky - isolated way obfuscation results.

Kaspersky’s detection rate for the original malware was 100%, as shown in Fig. 16. After applying 20 different obfuscation techniques, one after the other, the detection rate dropped to 55% (see Fig. 16). In addition, the obfuscation mechanisms such as try-catch, members-reordering, method-overloading, identifier-renaming, resource-renaming, string encryption and data encoding also have a significant impact on the detection rate of the Dr. Web anti-malware tool.

**Figure 17:**
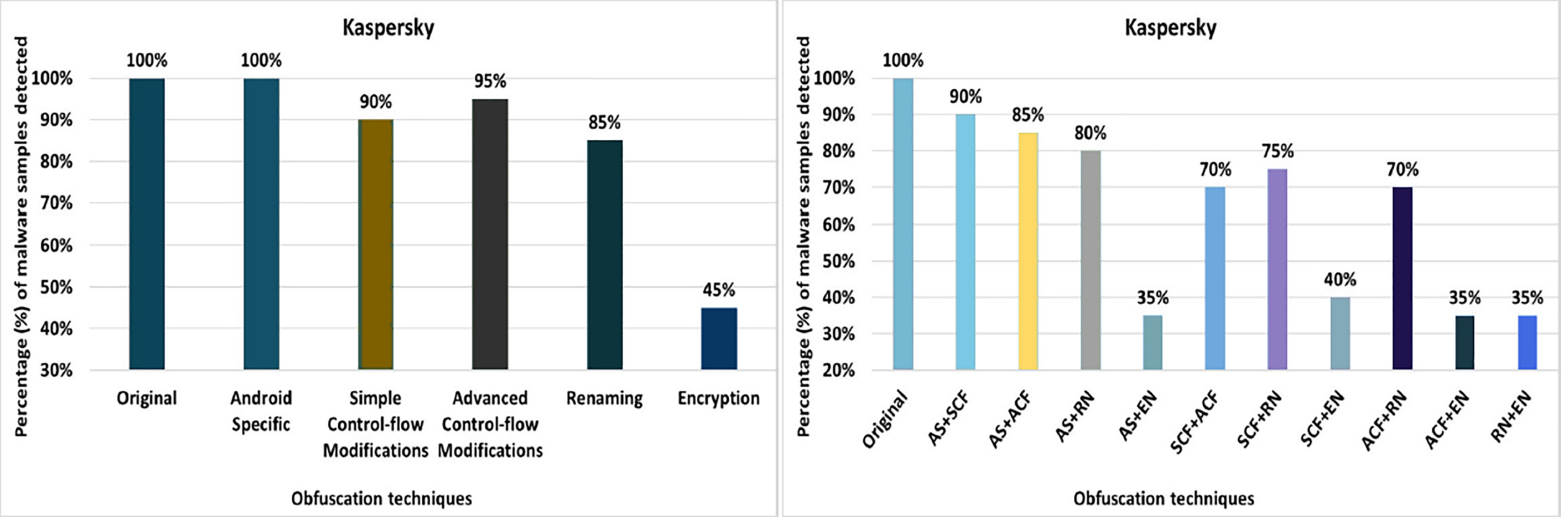
Kaspersky: category-wise and inter-category-wise results. (A) Kaspersky: category-wise obfuscation results. (B) Kaspersky: inter-category-wise obfuscation results.

When obfuscation techniques are applied in category-wise configurations, Kaspersky’s detection rate drops to 45%, as shown in Fig. 17A. In addition, the other obfuscation mechanisms also affect the detection rate, such as the categories control flow modifications, enhanced control flow modifications and renaming (see Fig. 17A). With inter-category obfuscation, the detection rate drops further to 35%. The notable inter-category obfuscation mechanisms that cause a low detection rate are Android-specific with encryption, simple control flow modifications along encryption, and advanced control flow modifications along encryption (as shown in Fig. 17B).

#### Malwarebytes security

**Figure 18:**
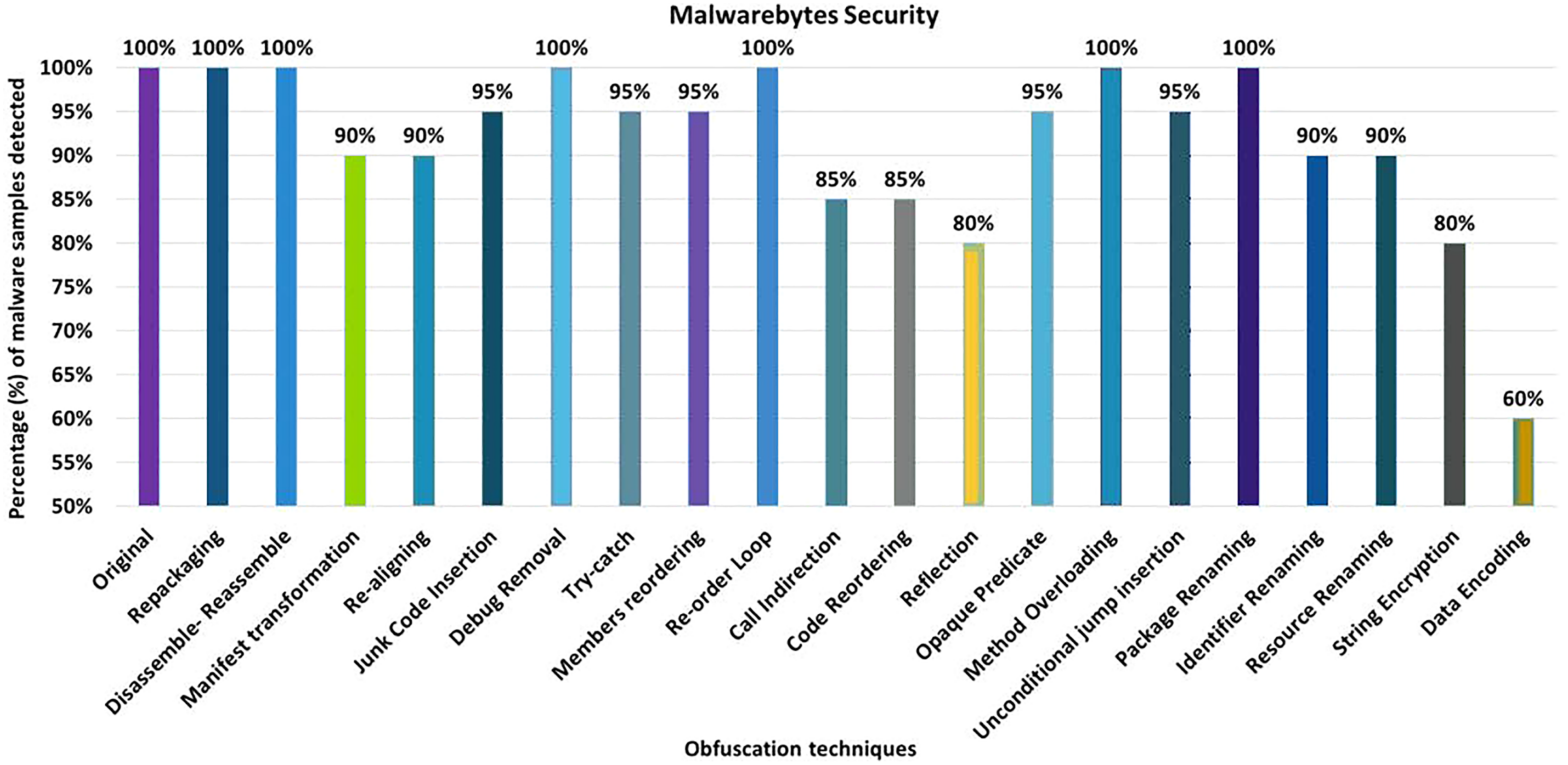
Malwarebytes: isolated way obfuscation results.

**Figure 19:**
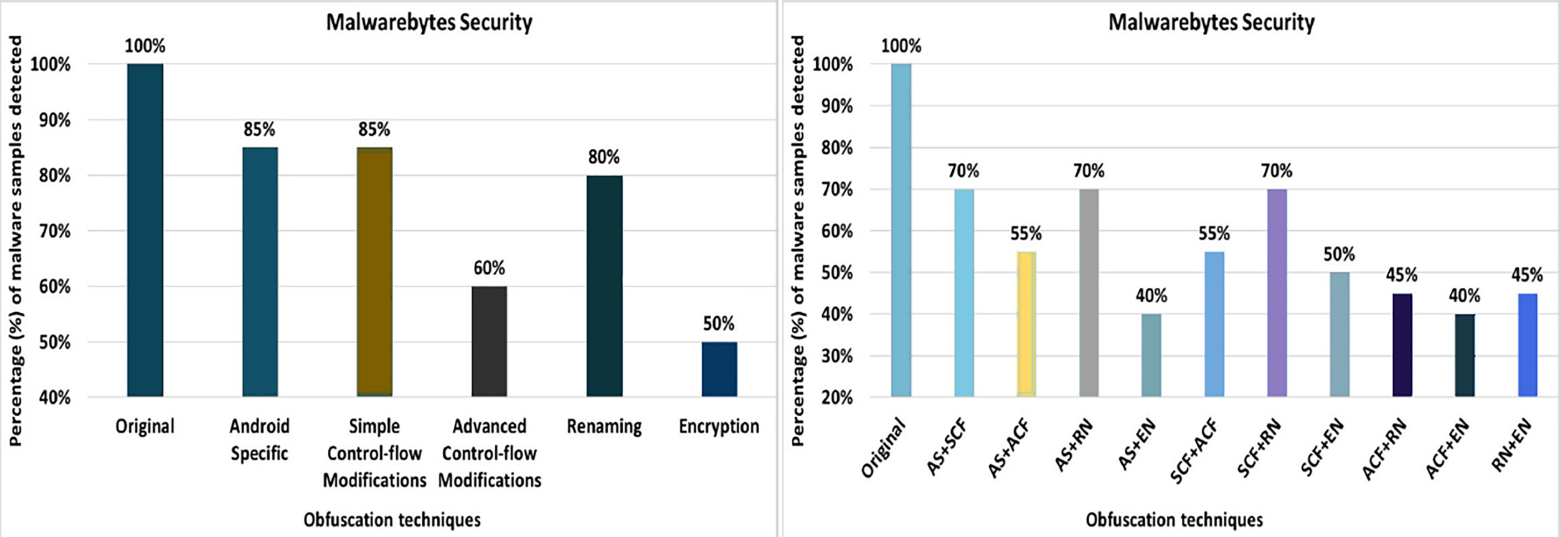
Malwarebytes: category-wise and inter-category-wise results. (A) Malwarebytes: category-wise obfuscation results. (B) Malwarebytes: inter-category-wise results.

The Malwarebytes anti-malware tool detects 100% of the original malware samples when analyzed against the raw malware samples. However, after applying the 20 obfuscation techniques, the detection rate dropped to 60%, which can be seen in Fig. 18. The effects of the different obfuscation techniques on the detection rate are shown in Fig. 18. When obfuscation techniques are applied in category-wise configurations, the detection rate increases to 50%, as shown in Fig. 19A. The results presented show that most of the obfuscation categories have a significant impact on the detection rate that can be found in Fig. 19A.

When obfuscation techniques are applied inter-category-wise, Malwarebytes’ detection rate drops to 40%, as shown in Fig. 19B. In addition to the Android-specific category with encryption, the other inter-category obfuscation techniques (*i.e*., advanced control flow modifications with encryption) also have a significant impact and reduced the detection rate to 40% that can be seen in Fig. 19B.

#### McAfee

**Figure 20:**
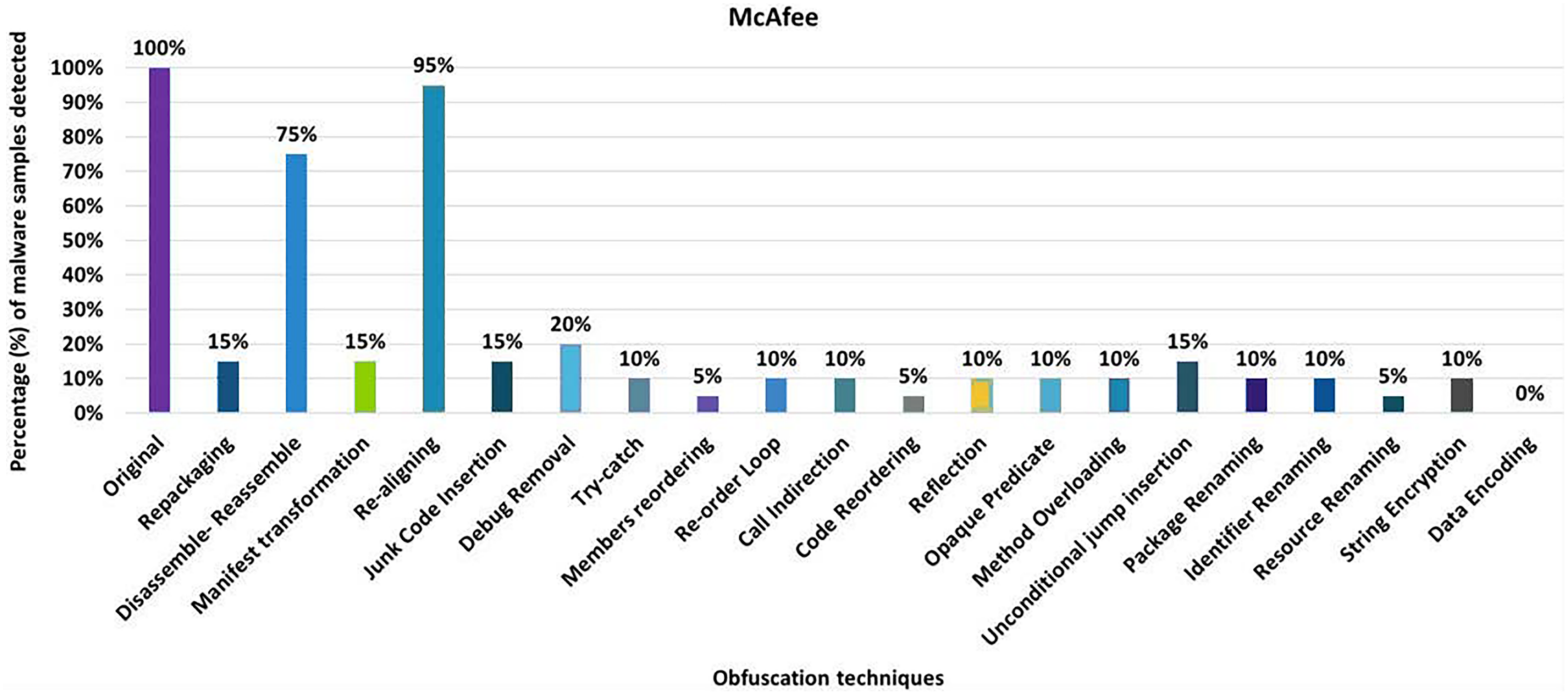
McAfee: isolated way obfuscation results.

McAfee’s anti-malware tool detected all the original malware samples as malicious. However, after applying 20 different obfuscation techniques (each separately), the detection rate dropped to as low as 0% for obfuscation data-encoding (as shown in Fig. 20). As Fig. 20 shows, the detection rate dropped significantly for the other obfuscation mechanisms in addition to data-encoding.

**Figure 21:**
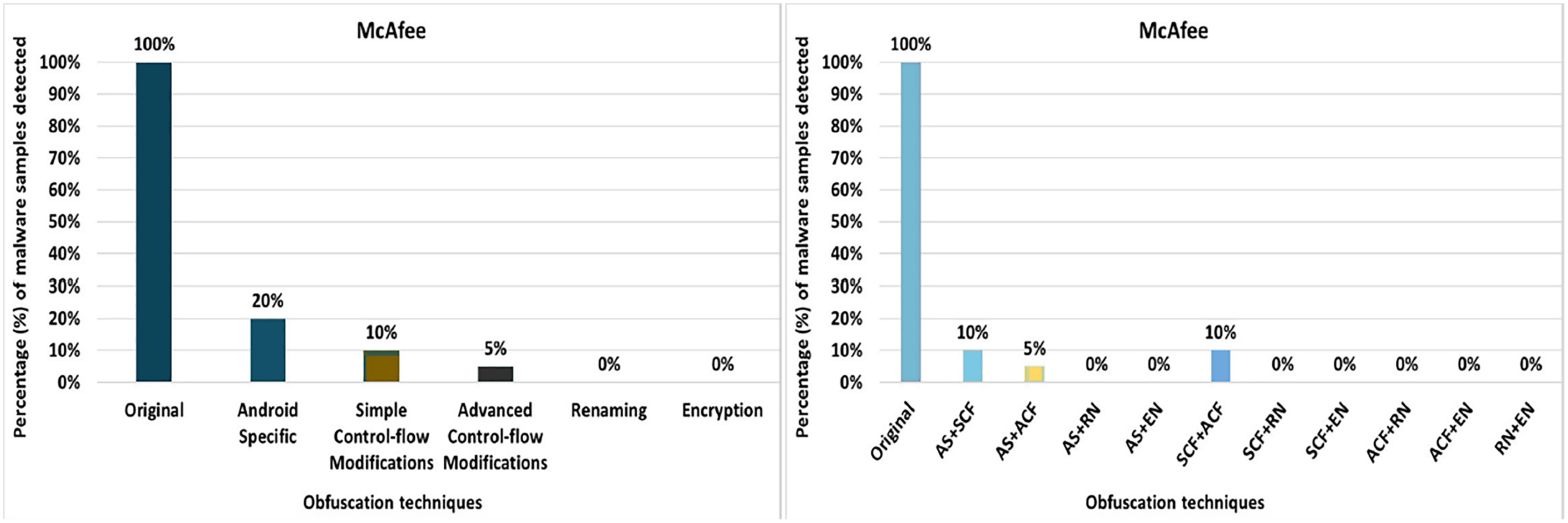
McAfee: category-wise and inter-category-wise results. (A) McAfee: category-wise obfuscation results. (B) McAfee: inter-category-wise obfuscation results.

When obfuscation techniques are applied category-wise, McAfee’s detection rate drops to as low as 0% (*i.e*., encryption along renaming obfuscation). As can be seen in Fig. 21A, all categories significantly affect the detection rate of the Malwarebytes anti-malware tool. The maximum detection rate with category-wise obfuscation is only 20%. These results show that combinations of obfuscation techniques significantly affect McAfee. When obfuscation techniques are applied inter-category-wise, McAfee’s detection rate also drops by 0% for most of the inter-category combinations that can be seen in Fig. 21B.

#### Sophos

**Figure 22:**
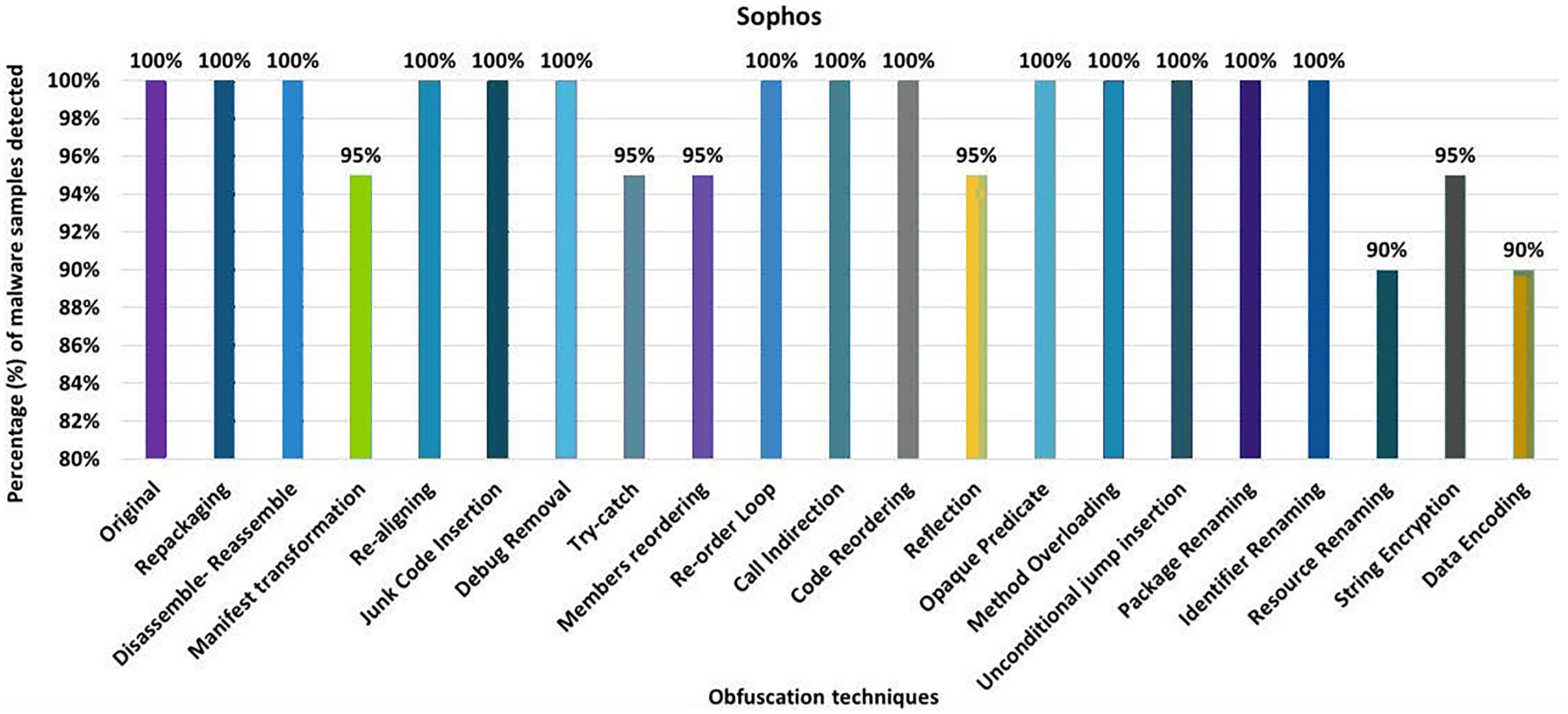
Sophos: isolated way obfuscation results.

**Figure 23:**
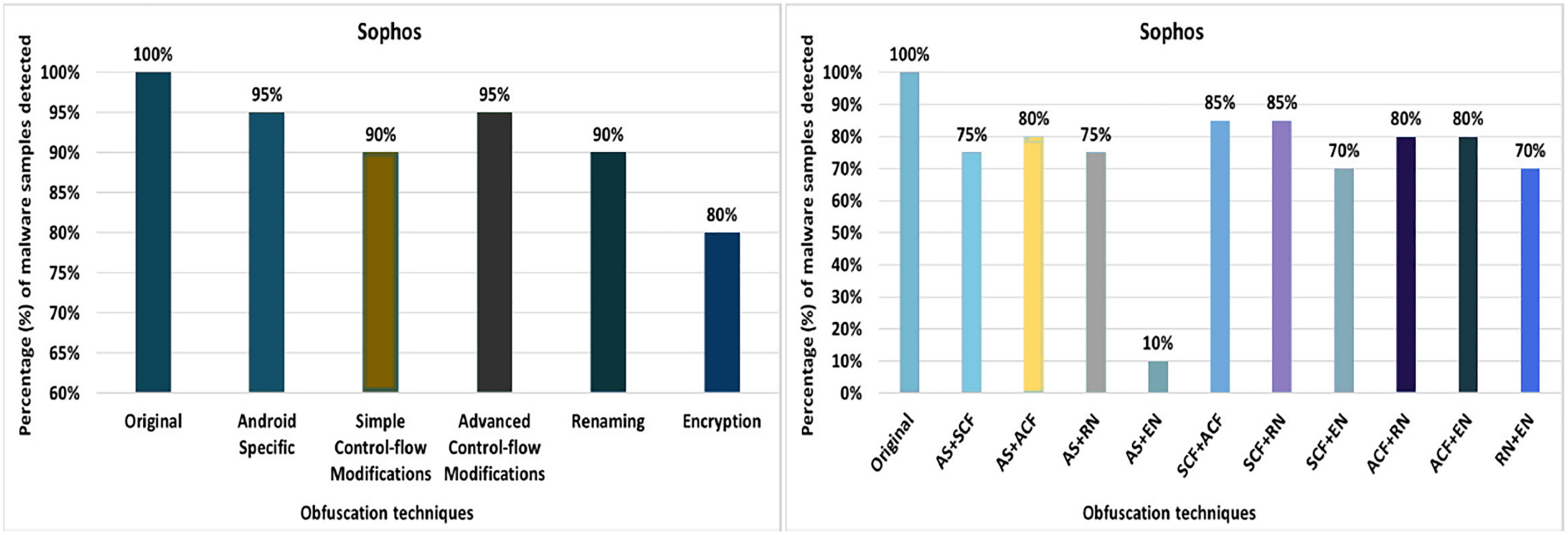
Sophos: category-wise and inter-category-wise results. (A) Sophos: category-wise obfuscation results. (B) Sophos: inter-category-wise obfuscation results.

Sophos’ anti-malware tool shows 100% detection of the raw malware samples. However, after applying 20 different obfuscation techniques (each separately), the detection rate drops to 90% (*i.e*., resource renaming and data encryption), as shown in Fig. 22. The results also show that Android-manifest-transformation, try-catch, members-reordering, reflection, and string-encryption also have notable effects on the detection rate of the Sophos anti-malware tool. When obfuscation techniques are applied on a category-wise, Sophos’ detection rate drops to 80% for the encryption category, as shown in Fig. 23A. The results show that all categories have an impact on the detection rate of Sophos’s anti-malware tool, with the Encryption category having the greatest impact, as shown in Fig. 23A. When using obfuscation categories, the maximum detection rate was 95%, and the minimum detection rate was 80%.

When tested using the inter-category obfuscation, Sophos’ detection rate dropped to just 10% (*i.e*., combining Android-specific with encryption category), as shown in Fig. 23B. In addition, the other inter-category obfuscations also resulted in a significantly lower detection rate.

### Results discussion

We use various obfuscation techniques applied to the well-known Android anti-malware tools in isolation, category-wise, and inter-category. Table 5 shows the summarized data related to the code obfuscation techniques used and the detection rates achieved. In general, the results show that obfuscation enhances the malware with stealth attributes. The results show that the more complicated the obfuscation mechanism is (*i.e*., inter-category obfuscations), the more likely the malware is to go undetected.

As shown in Table 7, Avira attains a detection rate of 100% when RP, DRe, RA, JCI, DebR, TC, ROL, CI, CR, Re, OP, MO, UJI, and PR are applied individually, but this rate drops to 85% with the obfuscation mechanism SE. When obfuscation techniques of the category EN are applied, this rate drops to 75%. In the case of an inter-category application, the detection rate drops even further, to 35%. Avast achieved the maximum detection rate of 80% with single obfuscation schemes, *e.g*., RP, DRe, AMT, RA, DebR, MO, PR, and RR. For avast, this detection rate drops further to 65% when the obfuscations SE and DE are used. The EN category shows the worst detection rate (*i.e*. 55%), which drops further to 35% when this obfuscation is combined with the AS category. In the AVG Mobile category, a maximum detection rate of 80% was achieved when the RP, DRe, AMT, RA, DebR, PR and RR obfuscation mechanisms were used. In the categories-wise analysis of AVG Mobile, the detection rate dropped to 50% for the category EN and 35% when the combination of the categories AS and EN was used. The Bitdefender anti-malware tool achieved a maximum detection rate of 100% for the individual obfuscation techniques. However, when the inter-category combination of AS and SCF was used, the detection rate dropped to 40%. In the case of Dr. Web, the maximum detection rate was 100% for the individual obfuscation techniques such as RP, DRe, AMT, RA, JCI, DebR, ROL, OP, UJI, PR, IR, and SE. For individual obfuscation categories, the detection rate was 100%, however, when multiple categories such as ACF and EN were combined, the detection rate dropped to 0% (*i.e*., all malicious applications escaped detection by Dr. Web). ESET Mobile Security has the lowest detection rate of 80% when single obfuscation techniques are used (such as Re); this rate drops further, *i.e*., to 70% when the ACF obfuscation category is applied; and to 50% when the combination of ACF and RN is used. In Kaspersky’s case, the worst detection rate is 55% when the obfuscation technique DE is applied individually. A detection rate of 45% is calculated for the category EN, when the obfuscation is used with a combination of two categories AS with EN, ACF with EN, and RN with EN, the results drop to 35%. The worst detection rate of Malwarebytes Security is 60% when DE is applied individually. Malware Security’s detection results dropped to 40% on inter-categories-wise combinations of AS with EN and ACF with EN.

**Table 7:** Tool-wise evaluation results.

Anti-Malware tools	Individual techniques		Category-wise techniques		Inter-category-wise techniques	
	Best	Worst	Best	Worst	Best	Worst
Avira	RP, DRe, RA, JCI, DebR, TC, ROL, CI, CR, Re, OP, MO, UJI, PR, (100%)	SE (85%)	AS, ACF (100%)	EN (75%)	AS+SCF, SCF+ACF, SCF+RN, ACF+RN, (90%)	AS+EN (35%)
Avast	RP, DRe, AMT, RA, DebR, MO, PR, RR, (80%)	SE, DE (65%)	AS (80%)	EN (55%)	AS+ACF, ACF+RN (75%)	AS+EN (35%)
AVG Mobile	RP, DRe, AMT, RA, DebR, PR, RR, (80%)	SE, DE (60%)	AS (80%)	EN (50%)	AS+SCF, AS+ACF, SCF+ACF, (75%)	AS+EN (30%)
Bitdefender	RP, DRe, AMT, MR, ROL, CI, CR, Re, MO, UJI, IR, RR, (100%)	RA,TC (80%)	ACF,EN (90%)	AS, SCF (75%)	ACF+RN, RN+EN (90%)	AS+SCF (40%)
Dr. Web	RP, DRe, AMT, RA, JCI, DebR, ROL, OP, UJI, PR, IR, SE, (100%)	CI, Re (40%)	AS (100%)	ACF (20%)	AS+SCF, AS+RN (90%)	ACF+EN (0%)
ESET Mobile Security	RP, DRe, AMT, RA, JCI, DebR, TC, OP, UJI, PR, IR (100%)	Re (80%)	AS (100%)	ACF (70%)	AS+RN (95%)	ACF+RN (50%)
Kaspersky	RP, DRe, AMT, RA, JCI, DebR, ROL, CI, CR, Re, OP, UJI, PR, (100%)	DE (55%)	AS (100%)	EN (45%)	AS+SCF (90%)	AS+EN, ACF+EN, RN+EN (35%)
Malwarebytes	RP, DRe, DebR, ROL, MO, PR, (100%)	DE (60%)	AS, SCF (85%)	EN (50%)	AS+SCF, AS+RN, SCF+RN, (70%)	AS+EN, ACF+EN (40%)
McAfee	RA (95%)	DE (0%)	AS (20%)	RN, EN (0%)	AS+SCF, SCF+ACF (10%)	AS+RN, AS+EN, SCF+RN, SCF+EN, ACF+RN, ACF+EN, RN+EN (0%)
Sophos	RP, DRe, RA, JCI, DebR, ROL, CI, CR, OP, MO, UJI, PR, IR, (100%)	RR, DE (90%)	AS, ACF (95%)	EN (80%)	SCF+ACF, SCF+RN (85%)	AS+EN (10%)

As shown in Table 8, a total of 700 obfuscated applications are generated using these obfuscation schemes to evaluate anti-malware tools. When 20 different obfuscation techniques are applied individually, the maximum average detection rate is 93.50% for disassembly and reassembly, while the minimum average detection rate is 70% for data-encryption related obfuscation. The worst detection rate is 0% for Data Encoding by McAfee.

**Table 8:** Technique-wise results.

Obfuscation technique	Best	Average	Worst
Repackaging	100%, (AVIRA, Bitdefender, Dr. Web, ESET, Kaspersky, Malwarebytes, Sophos)	87.50%	15%, (McAfee)
Disassemble-Reassemble	100%, (AVIRA, Bitdefender, Dr. Web, ESET, Kaspersky, Malwarebytes, Sophos)	93.50%	75%, (McAfee)
Manifest transformation	100%, (Bitdefender, Dr. Web, ESET, Kaspersky)	85%	15%, (McAfee)
Re-aligning	100%, (AVIRA, Dr. Web, ESET, Kaspersky, Sophos)	92.50%	80%, (Avast, AVG, Bitdefender)
Junk Code Insertion	100%, (AVIRA, Dr. Web, ESET, Kaspersky, Sophos)	85.50%	15%, (McAfee)
Debug Removal	100%, (AVIRA, Dr. Web, ESET, Kaspersky, Sophos, Malwarebytes)	87.50%	20%, (McAfee)
Try-catch	100%, (AVIRA, ESET)	81%	10%, (McAfee)
Members reordering	100%, (Bitdefender)	81.50%	5%, (McAfee)
Re-order Loop	100%, (AVIRA, Bitdefender, Dr. Web, Kaspersky, Malwarebytes, Sophos)	85%	10%, (McAfee)
Call Indirection	100%, (AVIRA, Bitdefender, Kaspersky, Sophos)	77%	10%, (McAfee)
Code Reordering	100%, (AVIRA, Bitdefender, Kaspersky, Sophos)	81%	5%, (McAfee)
Reflection	100%, (AVIRA, Bitdefender, Kaspersky)	75%	10%, (McAfee)
Opaque Predicate	100%, (AVIRA, Dr. Web, ESET, Kaspersky, Sophos)	83%	10%, (McAfee)
Method Overloading	100%, (AVIRA, Bitdefender, Malwarebytes, Sophos)	84%	10%, (McAfee)
Unconditional jump insertion	100%, (AVIRA, Bitdefender, Dr. Web, ESET, Kaspersky, Sophos)	86%	15%, (McAfee)
Package Renaming	100%, (AVIRA, Dr. Web, ESET, Kaspersky, Malwarebyte, Sopho)	86.50%	10%, (McAfee)
Identifier Renaming	100%, (Bitdefender, Dr. Web, ESET, Sophos)	83%	10%, (McAfee)
Resource Renaming	100%, (Bitdefender)	81.50%	5%, (McAfee)
String Encryption	100%, (Dr. Web)	74.50%	10%, (McAfee)
Data Encoding	95%, (AVIRA, Bitdefender, ESET)	70%	0%, (McAfee)
Obfuscation Categories			
Android Specific	100%, (AVIRA, Dr. Web, ESET, Kaspersky)	83.50%	20%, (McAfee)
Simple Control-flow Modifications	95%, (AVIRA)	76.50%	10%, (McAfee)
Advanced Control-flow Modifications	100%, (AVIRA)	68.50%	5%, (McAfee)
Renaming	95%, (Bitdefender, ESET)	77%	0%, (McAfee)
Encryption	90%, (Bitdefender)	61%	0%, (McAfee)
Obfuscation Inter-categories			
AS+SCF	90%, (AVIRA, Dr. Web, Kaspersky)	69.50%	10%, (McAfee)
AS+ACF	85%, (AVIRA, Kaspersky)	59.50%	5%, (McAfee)
AS+RN	95%, (ESET)	69.50%	0%, (McAfee)
AS+EN	80%, (ESET)	39.50%	0%, (McAfee)
SCF+ACF	90%, (AVIRA)	60%	10%, (McAfee)
SCF+RN	90%, (AVIRA)	66%	0%, (McAfee)
SCF+EN	75%, (Dr. Web)	50%	0%, (McAfee)
ACF+RN	90%, (AVIRA, Bitdefender)	57.50%	0%, (McAfee)
ACF+EN	85%, (Bitdefender)	46.50%	0%, (McAfee, Dr.Web)
RN+EN	90%, (Bitdefender)	55%	0%, (McAfee)

When the obfuscation techniques are applied category-wise, five categories emerged as shown in Table 4 (to evaluate anti-malware tools). The maximum average detection rate is 83.50% in the Android Specific category, while the minimum average detection rate is 61% in the Encryption category. The worst detection rate is 0% in McAfee’s Encryption category. The best detection rate is 100% in the Android Specific category by AVIRA, Dr. Web, ESET, and Kaspersky.

A significant effect on recognition was observed when two categories were combined. The maximum average recognition rate goes down to 69.50% for a variety of Android-specific categories with simple control flow and Android-specific categories with renaming, from 93.50% in single obfuscation cases and 83.50% for category-wise obfuscation schemes. The lowest average detection rate is 46.50% for a combination of advanced control-flow with renaming category, 70% in individual cases, and 61% for category-wise obfuscation schemes. The results also show the worst detection rate of 0% for 8 out of 10 inter-category-wise combinations. The best detection rate for inter-category combinations is 95% for the category Android-specific with renaming, which is detected by the ESET anti-malware tool.

McAfee has the worst detection rates of all anti-malware tools. McAfee’s best detection rate is 95% for the obfuscation technique RA. This rate drops to 0% with the single obfuscation technique DE. None of the anti-malware tools shows 0% with a single obfuscation technique except McAfee. It offers the best detection rate of 20% at AS, when the tool is evaluated by category. When inter-category combinations are applied, McAfee shows the best detection rate of 10% when combining AS with SCF and SCF with ACF. The results show a significant drop in McAfee’s detection rate (*i.e*., 0%) when inter-categories of AS with RN, AS with EN, SCF with RN, SCF with EN, ACF with RN, ACF with EN, and RN with EN. Sophos has a detection rate of 100% when RP, DRe, RA, JCI, DebR, ROL, CI, CR, OP, MO, UJI, PR, and IR are applied individually, but this rate drops by 90% with RR and DE. When obfuscation techniques of the category EN are used, this rate drops to 80%. In the case of the inter-category, this rate drops from 80% to 10% when the combination of AS and EN is applied.

## Conclusions and Future Work

In the past, researchers have evaluated anti-malware tools using various methods a limited number of and basic obfuscation techniques (Preda & Maggi, 2017; Hammad, Garcia & Malek, 2018; Bakour, Ünver & Ghanem, 2019; Chua & Balachandran, 2018; Balachandran et al., 2016). This work provides a comprehensive study based on a large number of obfuscation techniques applied individually, category-wise, and inter-category to evaluate prominent Android anti-malware tools. We use twenty different obfuscation techniques and applied them individually, category-wise, and inter-category-wise combinations to evaluate the top 10 commercially available prominent anti-malware tools. Five different categories are generated using the basic 20 obfuscation mechanisms. The evaluation results show that most of the malware tools could not able to detect malicious applications that have been obfuscated using multiple obfuscation techniques simultaneously (especially the inter-category wise combinations). The complex hybridization of obfuscation techniques used in this study provides a concerning insight into the weak detection capabilities of the prominent anti-malware tools against complex obfuscation mechanisms (*i.e*., inter-category wise obfuscations). In the future, we plan to evaluate these tools using more complex yet realistic obfuscation mechanisms by combining more inter-category-wise combinations.

## Additional Information and Declarations

### Data Availability

The following information was supplied regarding data availability: The data is available at GitHub: https://github.com/umairnawaz64/evaluation_of_antimalware_tools.
